# Beyond the Meat of the Matter: A Systematic Review and Meta-Analysis of the Hepatitis E Seroprevalence and Food-Borne Transmission Potential in the Balkans

**DOI:** 10.3390/v18070736

**Published:** 2026-07-02

**Authors:** Katerina Sakaliyska, Valeria Tonova, Hristo Manev, Tsvetoslav Koynarski, Georgi L. Lukov, Anton Andonov, Gergana Zahmanova

**Affiliations:** 1 Department of Molecular Biology, University of Plovdiv, 4000 Plovdiv, Bulgaria; 2 Center of Plant Systems Biology and Biotechnology, 4000 Plovdiv, Bulgaria; 3 Department of Medical Physics and Biophysics, Faculty of Pharmacy, Medical University of Plovdiv, 4002 Plovdiv, Bulgaria; 4 Research Institute, Medical University of Plovdiv, 4002 Plovdiv, Bulgaria; 5 Department of Animal Genetics, Faculty of Veterinary Medicine, Trakia University, 6000 Stara Zagora, Bulgaria; 6 Faculty of Sciences, Brigham Young University—Hawaii, Laie, HI 96762, USA; 7 Department of Medical Microbiology and Infectious Diseases, Max Rady College of Medicine, University of Manitoba, Winnipeg, MB R3T 2N2, Canada

**Keywords:** hepatitis E virus (HEV), seroprevalence, meta-analysis, Balkans, pork consumption, zoonosis, food-born transmission, One Health, blood donors, general population

## Abstract

Hepatitis E virus (HEV) is an emerging zoonotic pathogen in Europe, mainly transmitted via consumption of naturally contaminated food or contact with infected animals. People living in the Balkans have diverse dietary habits, with high pork consumption in some countries, making this region a relevant setting for investigating HEV seroprevalence and its possible determinants. The current study aimed to estimate pooled HEV seroprevalence among adults in the general population and blood donors and to assess factors associated with regional variation. Twenty-eight eligible studies were identified from PubMed, Scopus, and Web of Science following the PRISMA guidelines. Pooled prevalence estimates were calculated using a random-effects meta-analysis of proportions implemented via a generalized linear mixed model (GLMM) with logit transformation. Potential factors associated with HEV seroprevalence, including national pork consumption, serological assay type, population group, year of publication, sex, and country, were evaluated. The pooled anti-HEV seroprevalence was estimated to be 5.68% (95% CI: 3.48–9.12%), with substantial heterogeneity. Country-specific estimates ranged from 1.01% in Greece to 26.66% in Bulgaria. Subgroup analyses showed significant variation according to national pork consumption category, serological assay type, year of publication, and country. However, meta-regression indicated that methodological and temporal factors, particularly serological assay type and year of publication, were the main significant moderators, whereas national pork consumption was not independently associated with seropositivity. Therefore, pork consumption should be interpreted as an exploratory ecological indicator rather than as evidence of a direct association. The methodological differences contribute substantially to the variability in HEV seroprevalence across the Balkans, emphasizing the need for standardized diagnostic approaches within a One Health framework.

## 1. Introduction

Hepatitis E virus (HEV) is a significant global public health concern, mainly in regions with limited access to safe drinking water and sufficient sanitation [1,2]. In developed countries, zoonotic transmission has emerged as the predominant route of HEV infection, mainly through the consumption of undercooked pork and game meat or contact with infected animals [3,4,5]. Although HEV infection is often asymptomatic or self-limiting, it can cause severe complications such as acute liver failure and fulminant hepatitis. Pregnant women and immunocompromised individuals, including solid organ transplant recipients, are among the most vulnerable groups, with HEV infection often more severe in pregnant women and both acute and potentially chronic in immunocompromised individuals [6,7,8].

HEV is a small, quasi-enveloped, single-stranded, positive-sense RNA virus classified in the genus *Paslahepevirus* within the family *Hepeviridae* [9]. Two genera within this family infect mammals: *Paslahepevirus*, which includes strains that infect humans and different animal species, and *Rocahepevirus*, which comprises HEV strains primarily found in rodents and carnivores [10,11]. Among *Paslahepevirus balayani* species, there are eight genotypes (HEV-1 to HEV-8) [12]. Genotypes HEV-1 and HEV-2 are restricted to humans and transmitted predominantly via the fecal–oral route, typically causing large waterborne outbreaks in regions with poor sanitation, particularly in parts of Asia, Africa, and Central America [13]. In contrast, HEV-3 and HEV-4 are zoonotic, infecting humans and a range of animals, including domestic pigs, wild boars, deer, rabbits, goats, sheep, cows, horses, cats, and dogs [14,15,16]. These genotypes are the leading cause of autochthonous infections in high-income countries, transmitted mainly through the consumption of undercooked meat or direct contact with infected animals [17]. HEV-3 is widely distributed across Europe, North America, and parts of Asia, whereas HEV-4 is primarily confined to East Asia but is increasingly reported in Europe [18,19]. HEV-7, first identified in dromedary camels, has also been shown to infect humans. A documented case of chronic infection in a liver transplant recipient in the United Arab Emirates was linked to regular consumption of camel meat and milk [20]. HEV-8, also camel-associated, has not yet been confirmed to infect humans [21]. Genotypes HEV-5 and HEV-6, found in wild boars, have likewise not been linked to human infections [22,23]. Notably, *Rocahepevirus ratti* (rat HEV) has been identified as a cause of hepatitis in humans, particularly in immunocompromised individuals, indicating an emerging zoonotic threat with documented instances [24,25,26,27,28]. Furthermore, environmental detection of rat HEV was also reported [29].

In Europe, concern has grown over the increasing number of reports of locally acquired HEV-3 and HEV-4 infections [30,31]. However, it remains uncertain whether this reflects a genuine rise in HEV incidence or is a result of improved surveillance [6,32]. Reported anti-HEV IgG reactivity among blood donors in Europe varies considerably, ranging from 4.7% to 52.5% [33,34,35,36]. This substantial variation is influenced by factors such as donor age, sex, and assay type [37,38]. Although most HEV infections are asymptomatic, viremic blood donors may still pose a risk of transfusion-transmitted infection (TT-HEV), particularly for immunocompromised recipients and other vulnerable patients [39]. However, TT-HEV should be considered as one component of the broader HEV transmission landscape, which also includes food-borne exposure and direct or occupational contact with infected animals. Since universal HEV screening of blood donations is not uniformly mandatory across Europe, continuous epidemiological monitoring of HEV exposure and infection in blood donors remains important for risk assessment and for informing national blood safety policies [40]. Preventive measures are needed to reduce the global burden of both acute and chronic hepatitis E. Over the past 20 years, researchers have developed several candidate recombinant vaccines derived from different regions of the ORF2 capsid protein, which form virus-like particles (VLPs) that mimic the natural HEV surface. These ORF2 proteins have been produced in *E. coli*, insect cells, yeast, mammalian cells, and plants, and consistently show strong immune responses [41,42,43,44,45,46]. Although a recombinant HEV vaccine has been developed (Hecolin^®^), it is licensed and used in only a few countries [47].

Several systematic reviews on HEV seroprevalence have been published, each focusing on different geographical regions and populations. Hartl et al. (2016) and Aspinall et al. (2017) provided comprehensive analyses of anti-HEV IgG seroprevalence and HEV epidemiology across Europe [48,49,50]. However, neither study includes data from the Balkan countries. Wilhelm et al. (2019) conducted a systematic review and meta-analysis assessing HEV seroprevalence in the general population of non-endemic countries, while Li et al. (2020) presented a global synthesis of HEV epidemiology and associated risk factors [50,51]. Pavio et al. (2021) further conducted a systematic review and meta-analysis to explore risk factors for sporadic HEV infection [52]. Mrzljak et al. (2019, 2020) reviewed HEV epidemiology under the One Health framework in Eastern Europe, highlighting the zoonotic transmission of the virus [53,54]. Beyond Europe, Villalobos et al. (2022) estimated HEV seroprevalence across the Americas, and Mirzaev et al. (2024) assessed HEV seroprevalence patterns in Asia [55,56].

To date, no systematic review has specifically addressed the seroprevalence of HEV among blood donors and the general population in the Balkan region. The Balkans comprise a heterogeneous group of Southeastern European countries that differ in socioeconomic status, dietary habits such as pork consumption, and healthcare infrastructure [57]. Although individual seroprevalence studies have been conducted in several Balkan states, a comprehensive synthesis of the available evidence for the region as a whole has not yet been conducted. To address this gap, we conducted a systematic review and meta-analysis of HEV seroprevalence in the Balkans region. Using R, we performed random-effects meta-analyses and meta-regression models to generate pooled seroprevalence estimates and explore potential predictors, including country, diagnostic assay type, population age and sex structure, and study period. This approach provides a reliable quantitative assessment of HEV exposure across the region, facilitating a better understanding of epidemiological variability and key determinants of infection.

## 2. Materials and Methods

### 2.1. Eligibility Criteria

This systematic review and meta-analysis were conducted in accordance with the Cochrane Handbook for Systematic Reviews of Interventions and the PRISMA Statement [58]. The review protocol was not registered in PROSPERO or another publicly accessible registry. Ethical approval was not required, as only publicly available data from previously published studies were used. We included studies that met all of the following criteria: (1) published in indexed, peer-reviewed journals (full-text articles, letters, or correspondence with extractable original data); (2) reporting anti-HEV IgG and/or IgM seroprevalence data; (3) conducted in Balkan countries, defined as Albania, Bosnia and Herzegovina, Bulgaria, Croatia, Greece, Kosovo, Montenegro, North Macedonia, Romania, Serbia, Slovenia, and Turkey; (4) involving participants drawn from the general population or blood donors; and (5) including adults aged ≥18 years, or predominantly adult populations (≥15 years) when age-stratified data were unavailable and inclusion was unlikely to bias prevalence estimates.

For this review, the general population was defined as individuals recruited outside hospital settings and without known occupational exposure or high-risk medical conditions. Accordingly, the general population included non-remunerated blood donors, participants in community-based surveys, author-defined general population volunteers, and healthy control groups. We excluded controls recruited from hospital outpatient or inpatient settings, occupational cohorts (e.g., hunters, agricultural workers), and special populations, including pregnant women, children <15 years of age, and patients with chronic diseases. Studies without primary data (e.g., reviews, editorials) were also excluded. No language or time restrictions were applied (Table S1).

### 2.2. Search Strategy and Data Extraction

Exhaustive searches were conducted across the electronic databases PubMed, Scopus, and Web of Science, covering studies published before 15 January 2026. Although there is no universal agreement on what constitutes the Balkans, the following are usually included: Albania, Bosnia and Herzegovina, Bulgaria, Croatia, Greece, Kosovo, Montenegro, North Macedonia, Romania, Serbia, Slovenia, and Turkey. Based on this definition, the following primary search strategy was established: (Balkan* OR Albania OR “Bosnia and Herzegovina” OR Bulgaria OR Croatia OR Greece OR Kosovo OR Montenegro OR “North Macedonia” OR Romania OR Serbia OR Slovenia OR Turkey) AND (“Hepatitis E” OR HEV). It was further adapted for the different databases (Appendix A, Table A1). After reading the title and abstract, papers that did not address the seroprevalence of HEV in blood donors or the general population in the Balkan Peninsula were excluded from this systematic review. Articles with unclear titles and abstracts were read in full, and only those that contained the target content were included. Two independent investigators (K.T. and V.T.) screened the databases and extracted relevant information using Zotero. Differences in opinions about whether to include an article were resolved through discussion or by involving a third investigator (G.Z.).

### 2.3. Quality Assessment

The methodological quality of included studies was assessed using the Joanna Briggs Institute (JBI) Critical Appraisal Checklist for Studies Reporting Prevalence Data [59]. This tool evaluates nine domains related to sampling methods, study design, measurement validity, and statistical analysis. Each item was rated as “yes”, “no”, “unclear”, or “not applicable”. When studies reported multiple populations, only the subgroup meeting the inclusion criteria (e.g., blood donors) was extracted and considered for risk-of-bias assessment. The results of the quality assessment are summarized in Appendix A, Table A2.

### 2.4. Statistical Analysis

All statistical analyses were performed in R (version 4.4.0; R Foundation for Statistical Computing, Vienna, Austria) using RStudio (version 4.3.3, 29 February 2024). Meta-analyses, heterogeneity statistics, forest plots, and sensitivity analyses were conducted using the meta package (version 8.3-0). Statistical significance was defined as a two-sided *p*-value < 0.05.

#### 2.4.1. Effect Size and Pooling Method

The primary outcome was anti-HEV seroprevalence (IgG and/or IgM), expressed as the proportion of seropositive individuals among the total number tested in each study. Pooled prevalence estimates were calculated using a random-effects meta-analysis of proportions implemented via a generalized linear mixed model (GLMM) with logit transformation (sm = “PLOGIT”, method = “GLMM”). This approach directly models the binomial distribution of events, and study weights are not displayed because pooling is based on binomial likelihood rather than inverse-variance weighting. It is appropriate for prevalence data, including studies with low event rates and varying sample sizes. By inherently accommodating studies with zero events, the GLMM approach avoids the need for continuity corrections and the associated biases of transformation-based methods. Results were reported as pooled proportions (%) with corresponding 95% confidence intervals (CIs).

Because blood donors and the general population may differ in health status, selection mechanisms, and exposure risk, the pooled estimate represents a composite of distinct cohorts. Therefore, it should not be viewed as a singular parameter for a uniform target population, but rather as a synthesis of heterogeneous groups.

To evaluate robustness, alternative random-effects models were explored in sensitivity analyses, including the Freeman–Tukey double-arcsine transformation (sm = “PFT”, method = “Inverse”) and the inverse-variance logit model (sm = “PLOGIT”, method = “Inverse”). Estimates derived from alternative pooling methods were compared with the primary GLMM for pooled prevalence, precision (95% CIs), and heterogeneity measures (τ^2^ and I^2^) to assess the stability and robustness of the overall estimate.

#### 2.4.2. Heterogeneity and Sensitivity Analysis

Between-study heterogeneity was assessed using Cochran’s Q test, the between-study variance (τ^2^), and the inconsistency statistic (I^2^), which quantifies the proportion of total variability attributable to true between-study heterogeneity rather than sampling error. Heterogeneity was interpreted using conventional thresholds, with values of approximately 25%, 50%, and 75% indicating low, moderate, and high heterogeneity, respectively.

To make the data easier to interpret despite significant differences between studies, 95% prediction intervals were calculated to represent the expected range of true seroprevalence in a new study setting.

A leave-one-out (LOO) sensitivity analysis was conducted to assess the influence of individual studies on the pooled estimate. In this procedure, each study was sequentially excluded and the pooled prevalence recalculated. Changes in the pooled estimate (τ^2^) and I^2^ were examined to identify potentially influential studies and evaluate the robustness of the findings.

#### 2.4.3. Subgroup and Meta-Regression Analysis

Potential sources of heterogeneity were further explored by subgroup analysis and meta-regression. Subgroup analyses were conducted for: (1) pork consumption level, categorized as very low, low, medium, or high according to national per-capita pork consumption data (FAOSTAT); (2) serological assay type; (3) population type (general population vs. blood donors); (4) year of publication (<2000, 2000–2010, >2010); (5) sex, for studies reporting male and female data separately; and (6) country. Differences between subgroups were tested using χ^2^ tests in the meta package.

To investigate potential sources of between-study heterogeneity, mixed-effects meta-regression models were fitted using study-level covariates. The analyses were performed using the metareg function from the package meta (version 4.3.3). Continuous moderators included year of publication, national pork consumption (kg per capita per year), and mean participant age. For studies reporting median and range instead of mean and standard deviation, mean values were estimated using the quantile estimation methods of Luo et al. (2018) and Wan et al. (2014), as implemented in the R package (version 4.4.0) estmeansd, version 4.3.3, 29 February 2024 [60,61]. Also, when studies reported separate subgroup means (e.g., by sex or occupation), the overall mean age was recalculated by deriving a weighted mean based on subgroup sample sizes. Categorical moderators included serological assay type and population group (blood donors vs. general population). Assay type was entered as a categorical variable. While the Abbott ELISA served as the initial reference category due to its historical use, temporal changes in assay performance and market availability mean that comparisons between assays may be partially confounded by study period and should be interpreted with caution. For studies reporting sex-stratified seroprevalence, a multilevel meta-regression model was applied to account for within-study clustering of male and female strata. In this model, sex was included as a moderator, and the study identifier was specified as a random effect to account for correlations within strata originating from the same study.

Regression coefficients (β) were estimated on the logit scale, consistent with the primary generalized linear mixed model. Where appropriate, regression coefficients were also expressed as odds ratios to facilitate interpretation of effect sizes. Statistical significance was evaluated using Wald-type tests, and the overall contribution of categorical moderators was assessed using omnibus tests of moderators (QM statistics). Model parameters were estimated using maximum likelihood (ML). Residual heterogeneity in mixed-effects meta-regression models was quantified using the between-study variance (τ^2^) and the proportion of variability attributable to heterogeneity (I^2^). For the multilevel sex model, between-study variance was estimated using σ^2^, and residual heterogeneity was evaluated using the QE statistic.

#### 2.4.4. Publication Bias

Publication bias was assessed using the Doi plot and the Luis Furuya–Kanamori (LFK) index, which have been recommended as more appropriate methods than conventional funnel plots for detecting asymmetry in meta-analyses of proportions [62]. The LFK index was interpreted using established thresholds: values within ±1 indicating no asymmetry, values between ±1 and ±2 indicating minor asymmetry, and values exceeding ±2 indicating major asymmetry. Because asymmetry in prevalence meta-analyses may arise from sources other than publication bias, including substantial between-study heterogeneity and methodological differences, the Doi plot and LFK index were interpreted as measures of small-study effects rather than definitive evidence of publication bias.

## 3. Results

### 3.1. Study Selection and Characteristics

**Figure 1:**
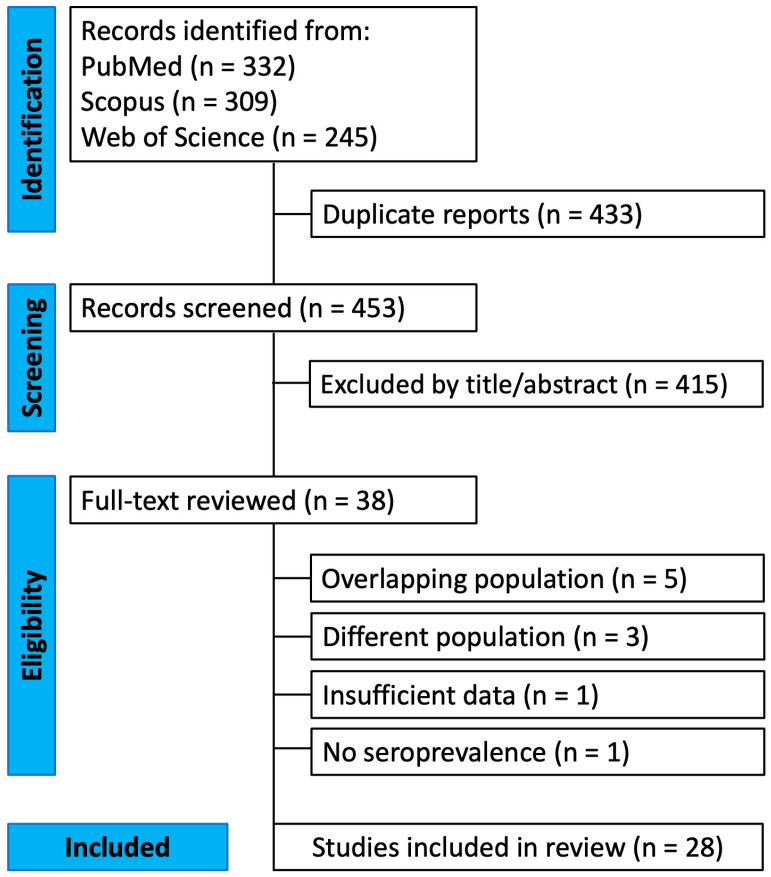
PRISMA flow diagram of study screening and selection.

A total of 886 studies were identified across PubMed (*n* = 332), Scopus (*n* = 309), and Web of Science (*n* = 245). After removing duplicate articles from the databases (*n* = 433), unrelated records were also excluded after title and abstract screening (*n* = 415). In total, 38 articles were read in full, and further application of inclusion and exclusion criteria identified 28 papers potentially suitable for the systematic review, yielding a nonoverlapping population of 13,399 individuals (Figure 1). The main characteristics of the individual studies are presented in Table 1. The list of studies reviewed in full and excluded is available in Appendix A, Table A3.

### 3.2. Overall Pooled HEV Seroprevalence

**Figure 2:**
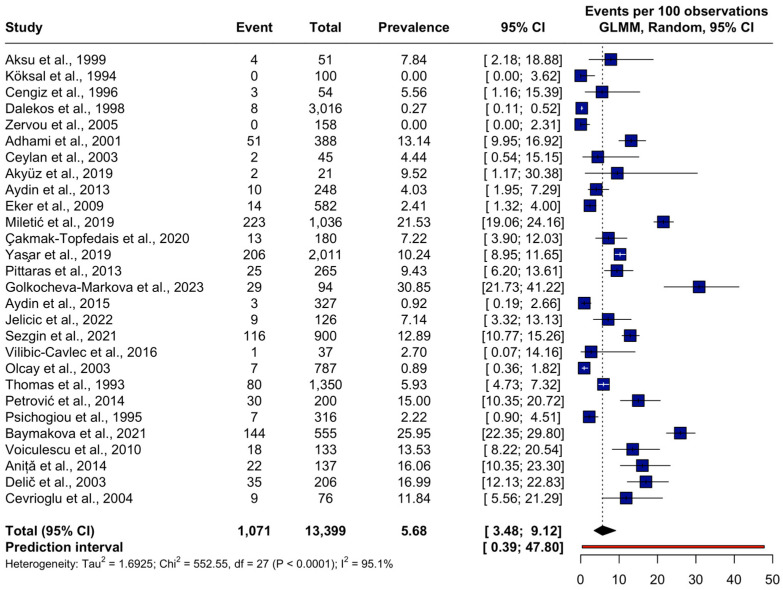
Random-effects forest plot showing anti-HEV seroprevalence in the general population and blood donors across 28 studies conducted in the Balkans. Individual study estimates are presented as proportions (events/total) with corresponding 95% confidence intervals (CIs). Squares represent study-specific prevalence estimates, and horizontal lines indicate 95% CIs. In this framework, study weights are not displayed because pooling is based on binomial likelihood rather than inverse-variance weighting. The final row presents the overall pooled estimate across all included studies, with the pooled prevalence (diamond) estimated using a generalized linear mixed model with logit transformation (PLOGIT–GLMM). The width of the diamond corresponds to the 95% CI of the pooled estimate. Between-study heterogeneity was assessed using Cochran’s Q test and quantified with Tau^2^ (between-study variance) and I^2^, which reflects the proportion of total variability attributable to true between-study heterogeneity rather than sampling error.

The pooled anti-HEV seroprevalence across all 28 eligible studies was estimated using a random-effects model with logit transformation (PLOGIT-GLMM). The overall prevalence among adults from the general population and blood donors across the Balkans was 5.68% (95% CI: 3.48–9.12%), indicating substantial between-study heterogeneity (I^2^ = 95.1%, τ^2^ = 1.69, *p* < 0.0001; Figure 2). Reported seroprevalence varied widely across countries, ranging from 0% in Turkey and Greece to 31% in Bulgaria.

**Figure A1:**
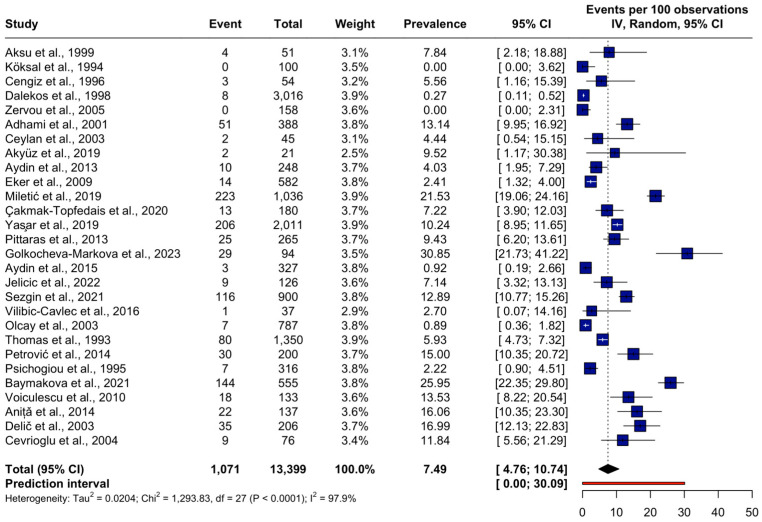
Overall pooled HEV seroprevalence using the Freeman–Tukey double arcsine transformation (PFT + Inverse variance model). Forest plot showing the pooled anti-HEV seroprevalence across 28 studies using the Freeman–Tukey (PFT) transformation with inverse-variance pooling. The pooled prevalence was 7.49% (95% CI 4.76–10.74), with high between-study heterogeneity (I^2^ = 97.9%, τ^2^ = 0.0204, *p* < 0.001). The result was consistent in direction and magnitude with the main PLOGIT–GLMM model (5.68%; Figure 2), confirming the robustness of pooled estimates to transformation choice.

**Figure A2:**
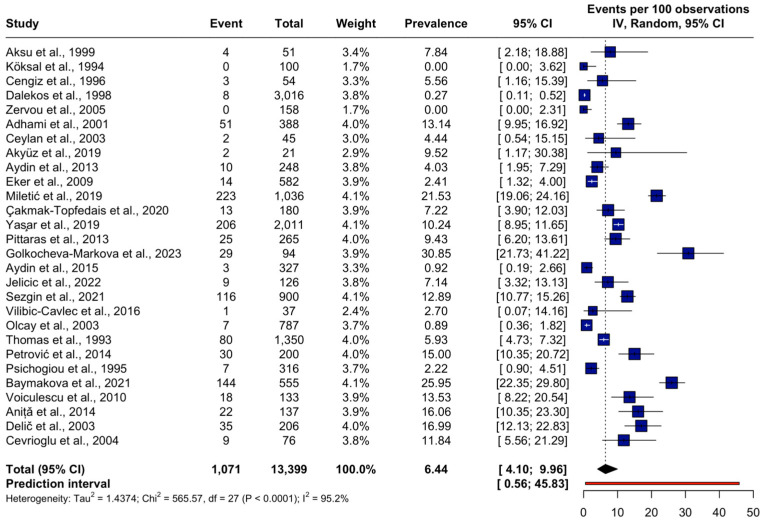
Overall pooled HEV seroprevalence using logit transformation with inverse-variance pooling (PLOGIT + Inverse model). Forest plot of the pooled anti-HEV seroprevalence under a logit transformation with inverse-variance weighting. The estimated pooled prevalence was 6.44% (95% CI 4.1–9.96), with substantial heterogeneity (I^2^ = 95.2%, τ^2^ = 1.44, *p* < 0.001). These findings are nearly identical to those obtained with the PLOGIT–GLMM approach (Figure 2), demonstrating stability across alternative pooling methods.

Alternative pooling methods were used to assess the robustness of the overall HEV seroprevalence estimates. The Freeman–Tukey double arcsine transformation with an inverse-variance random-effects model was used, and the pooled seroprevalence was 7.49% (95% CI: 4.76–10.74), while the logit transformation with inverse-variance weighting yielded a slightly lower seroprevalence of 6.44% (95% CI: 4.10–9.96). Despite differences in point estimates and between-study variance, the results remained broadly consistent with the primary GLMM-based analysis, indicating that the choice of pooling method did not drive the overall findings. Substantial heterogeneity persisted across all models (I^2^ > 95%); see Figure A1 and Figure A2.

### 3.3. Subgroup Analysis

Because dietary exposure is a key route of zoonotic HEV-3 transmission and pork consumption varies substantially across the Balkan region, we conducted a subgroup analysis stratifying studies by national per-capita pork consumption (Table 2). National pork consumption was treated as an ecological, study-level covariate based on the most recent available country-level estimates, reflecting population-level dietary exposure rather than individual intake. This approach allowed us to examine whether differences in dietary habits might partly explain the observed between-study heterogeneity and whether pork consumption was associated with HEV seroprevalence.

Serological assays were grouped by assay manufacturer and testing platform to account for methodological heterogeneity in antigen composition, analytical performance, and case definition across ELISA kits. Assays marketed under different regional entities of the same manufacturer were considered equivalent; however, to ensure sufficient statistical power, assays used infrequently were grouped as “other commercial ELISA”, while in-house or insufficiently described assays were analyzed as a separate category. In addition, subgroup analyses by population type (general population vs. blood donors) were conducted to evaluate potential differences related to participant selection, health status, and screening practices inherent to donor-based studies. Studies were further stratified by year of publication (<2000, 2000–2010, >2010) to explore temporal variation in HEV seroprevalence that may reflect changes in exposure patterns, diagnostic practices, or surveillance over time. Moreover, subgroup analyses by sex were performed to assess potential differences in HEV seroprevalence between males and females, given previously reported sex-related differences in exposure patterns and occupational or behavioral risk factors; sex-stratified data were extracted as reported by the original studies and were not available for all datasets. Finally, subgroup analyses by country were conducted to account for geographic heterogeneity across the Balkan region and to evaluate country-specific pooled seroprevalence estimates in relation to differences in epidemiological context, dietary habits, and study characteristics. Subgroup results are summarized in Table 3.

Subgroup analyses demonstrated significant variation in seropositivity according to pork consumption category, diagnostic assay, year of publication, and geographic location. The highest pooled anti-HEV seropositivity was observed in the high pork-consumption category (13.99%, 95% CI: 10.28–18.74), followed by the very low/low category (4.71%, 95% CI: 2.85–7.68), whereas the moderate category showed the lowest pooled estimate (3.33%, 95% CI: 0.53–18.33; *p* for subgroup differences = 0.0005) (Table 3).

Seroprevalence estimates varied significantly by serological assay (*p* = 0.0011). The highest pooled estimates were observed for Mikrogen ELISA (19.86%, 95% CI: 12.36–30.35) and other commercial ELISA assays (12.75%, 95% CI: 8.24–19.21), followed by Dia. Pro ELISA (6.70%, 95% CI: 3.08–13.96) and Euroimmun ELISA (4.19%, 95% CI: 1.36–12.16), while lower estimates were reported for Genelabs ELISA (2.39%, 95% CI: 0.61–8.91) and Abbott ELISA (1.25%, 95% CI: 0.19–7.57) (Table 3).

**Figure 3:**
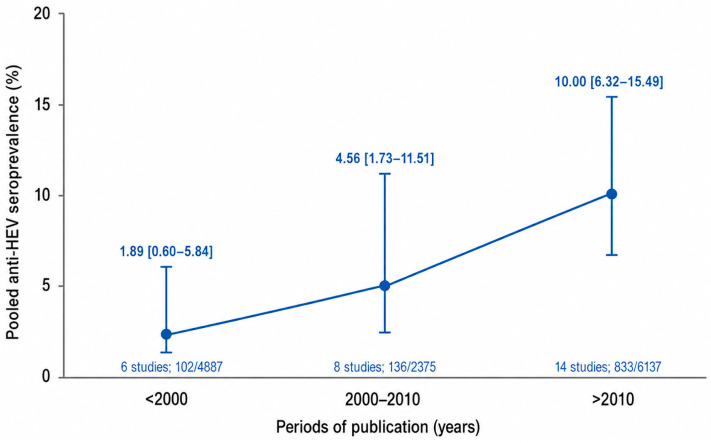
The prevalence of anti-HEV total immunoglobulins across the Balkans throughout the decades.

HEV seroprevalence was higher among blood donors (9.70%, 95% CI: 5.06–17.79) compared with the general population (4.46%, 95% CI: 2.40–8.14), although this difference did not reach statistical significance (*p* = 0.0854) (Table 3). When stratified by year of publication, seroprevalence was lowest in studies conducted before 2000 (1.89%, 95% CI: 0.60–5.84), increased during 2000–2010 (4.56%, 95% CI: 1.73–11.51), and was highest in studies conducted after 2010 (10.00%, 95% CI: 6.32–15.49) (Figure 3). The difference across publication periods was statistically significant (*p* = 0.0155) (Table 3).

**Figure 4:**
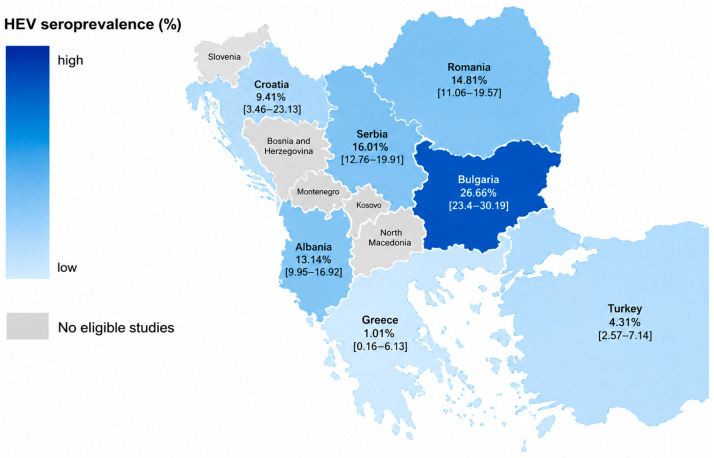
Polled anti-HEV total immunoglobulins among the general population and blood donors across Balkan countries.

Pooled seroprevalence estimates were comparable between males (5.96%, 95% CI: 2.88–11.91) and females (7.97%, 95% CI: 5.30–11.80), with no significant subgroup difference (*p* = 0.4843). Marked differences were observed between countries (*p* < 0.0001). The highest pooled seroprevalence was reported in Bulgaria (26.66%, 95% CI: 23.40–30.19) and Serbia (16.01%, 95% CI: 12.76–19.91), followed by Romania (14.81%, 95% CI: 11.06–19.57) and Croatia (9.41%, 95% CI: 3.46–23.13). Lower pooled estimates were observed in Turkey (4.31%, 95% CI: 2.57–7.14) and Greece (1.01%, 95% CI: 0.16–6.13). Figure 4 summarizes the data from Table 3 for pooled HEV seroprevalence in the Balkans.

**Figure A3:**
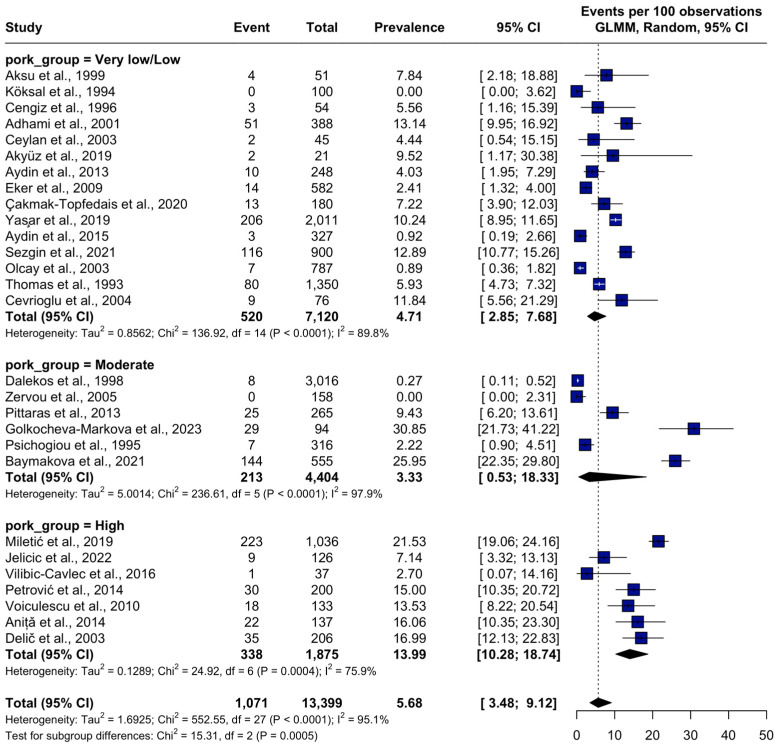
Forest plot of pooled HEV seroprevalence stratified by national pork consumption category (very low/low, moderate, high). Pooled estimates were calculated using a random-effects generalized linear mixed model (GLMM) with logit transformation. Squares represent individual study estimates with 95% confidence intervals (CIs); diamond shapes indicate pooled subgroup estimates. For Anițǎ et al., 2014, only data from the 2012 cohort were included [73]. For Voiculescu et al., 2010, only data from the healthcare professionals cohort were included [74].

**Figure A4:**
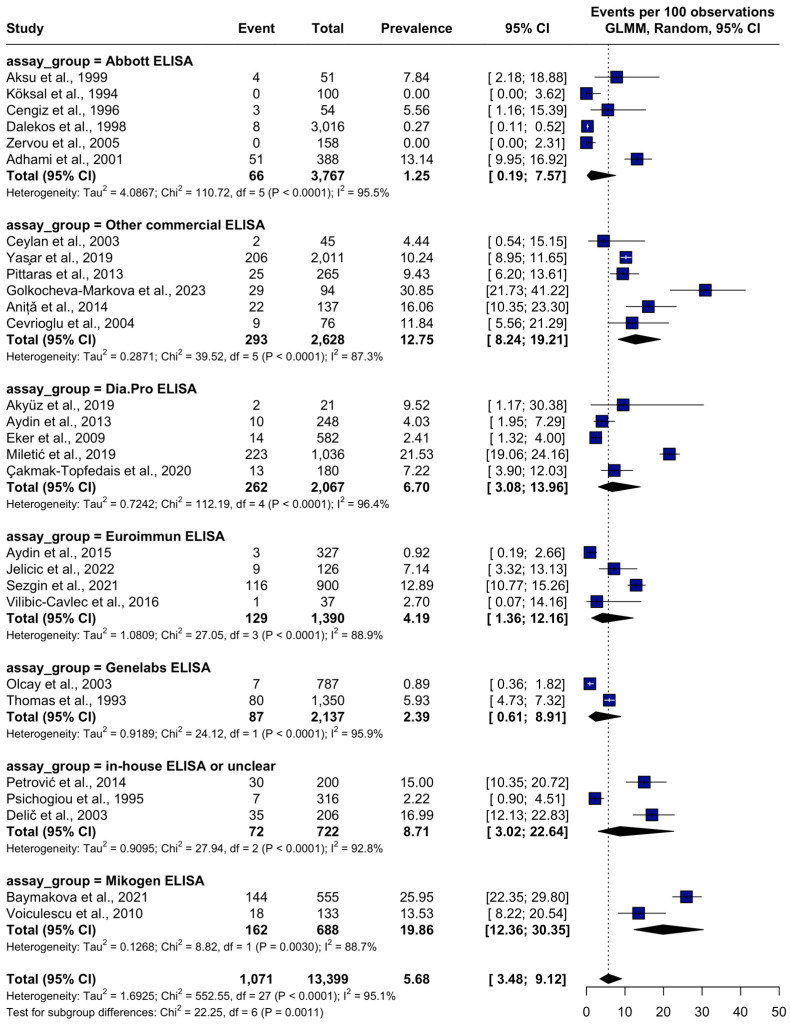
Forest plot of pooled HEV seroprevalence stratified by serological assay group (Abbott ELISA, Dia.Pro ELISA, Euroimmun ELISA, Mikrogen ELISA, Genelabs ELISA, other commercial ELISA, and in-house/unclear assays). Pooled estimates were calculated using a random-effects GLMM with logit transformation. Squares represent individual study estimates with 95% CIs; diamonds indicate pooled subgroup estimates.

**Figure A5:**
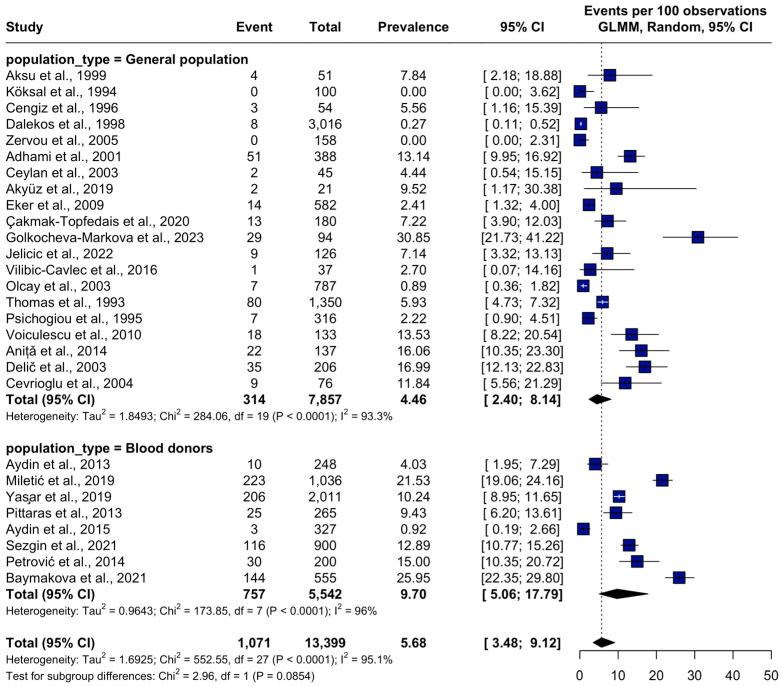
Forest plot of pooled HEV seroprevalence stratified by population type (general population vs. blood donors). Pooled estimates were calculated using a random-effects GLMM with logit transformation. Squares represent individual study estimates with 95% CIs; diamonds indicate pooled subgroup estimates.

**Figure A6:**
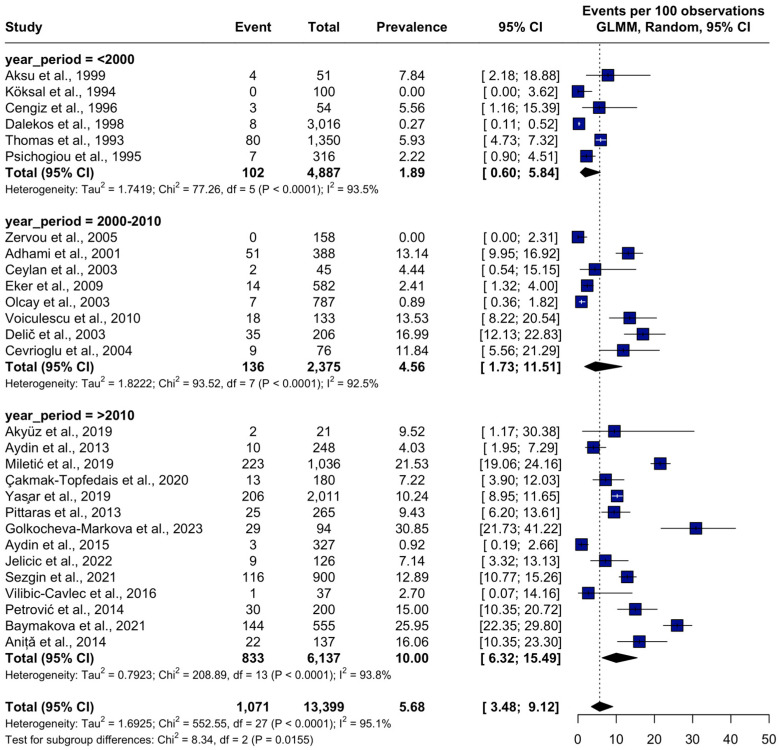
Forest plot of pooled HEV seroprevalence stratified by study period (<2000, 2000–2010, >2010). Pooled estimates were calculated using a random-effects GLMM with logit transformation. Squares represent individual study estimates with 95% CIs; diamonds indicate pooled subgroup estimates.

**Figure A7:**
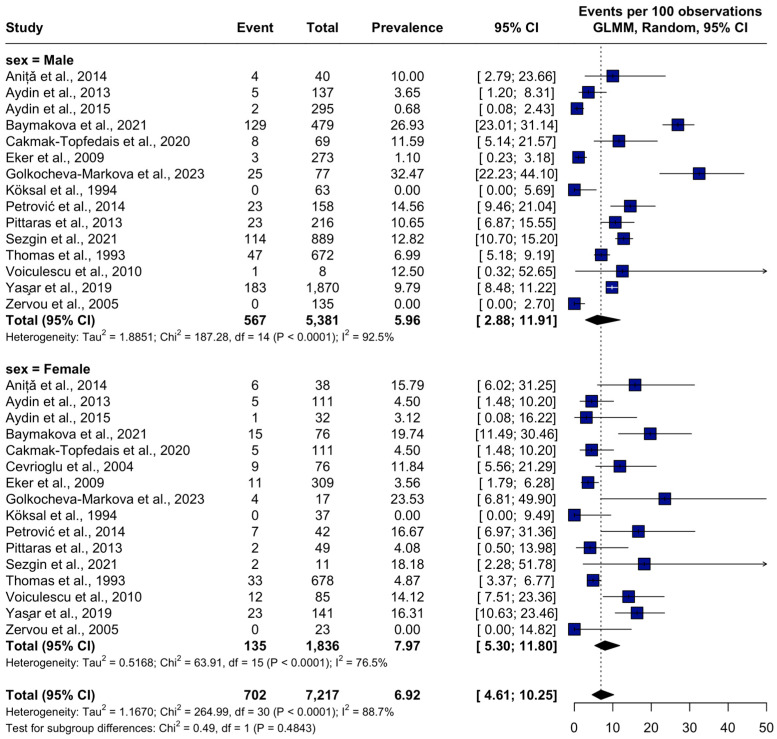
Forest plot of pooled HEV seroprevalence stratified by sex (male vs. female). Pooled estimates were calculated using a random-effects GLMM with logit transformation. Squares represent individual study estimates with 95% CIs; diamonds indicate pooled subgroup estimates.

**Figure A8:**
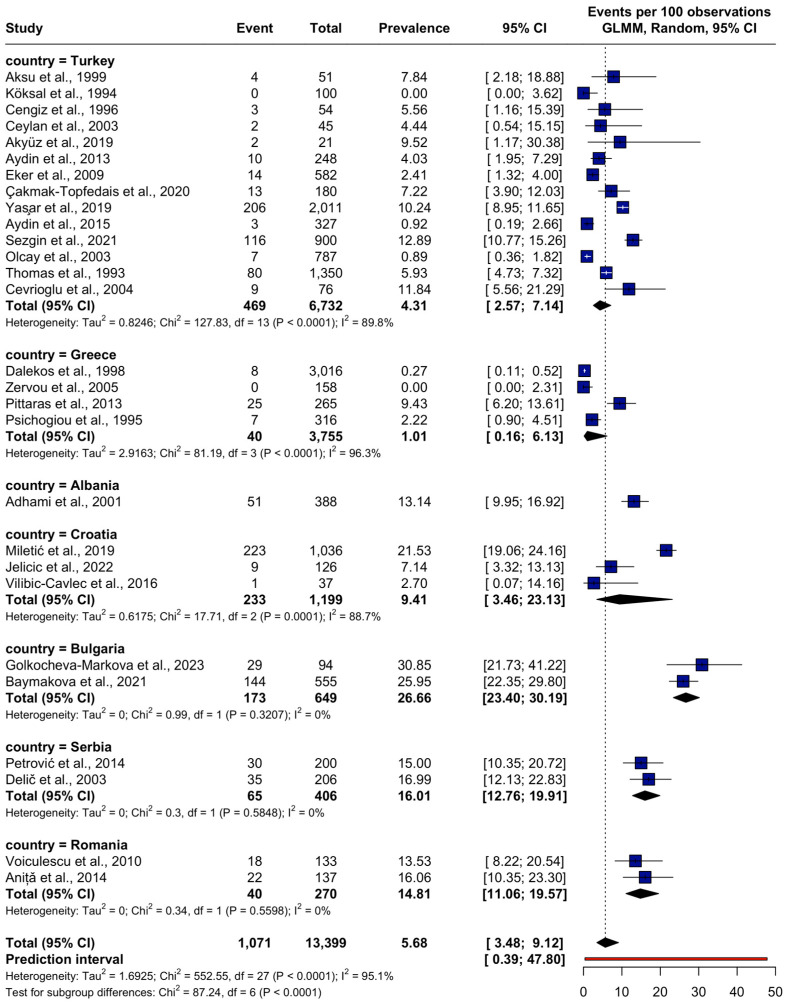
Forest plot of pooled HEV seroprevalence stratified by country. Pooled estimates were calculated using a random-effects GLMM with logit transformation. Squares represent individual study estimates with 95% CIs; diamonds indicate pooled subgroup estimates.

Substantial heterogeneity remained in all subgroup analyses. The corresponding forest plots are provided in Appendix A (Figure A3, Figure A4, Figure A5, Figure A6, Figure A7 and Figure A8).

### 3.4. Meta-Regression

**Figure 5:**
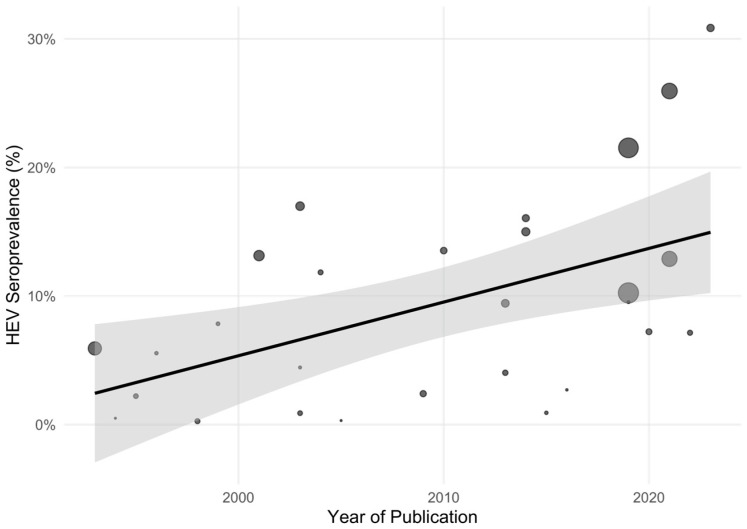
Bubble plot showing the association between year of publication and reported HEV seroprevalence in the Balkans (univariable model, k = 28). Bubble size is proportional to study precision (inverse-variance weighting). The regression is performed on logit-transformed prevalence estimates. The solid line represents the fitted random-effects meta-regression model, with the shaded area indicating the 95% confidence interval.

To further investigate potential sources of between-study heterogeneity, mixed-effects meta-regression analyses were conducted using study-level covariates (Table A3). In univariable analyses including all 28 studies, year of publication was significantly associated with HEV seroprevalence (β = 0.0716, *p* = 0.0025), indicating higher reported prevalence in more recently published studies (Figure 5). National pork consumption (kg/person/year), included as an ecological study-level covariate, showed a positive but non-significant association with seroprevalence (β = 0.0180, *p* = 0.1012). Thus, although subgroup analysis suggested differences across pork-consumption categories, this ecological variable was not significantly associated with anti-HEV seropositivity in univariable meta-regression and was not an independent predictor in the multivariable model.

In a multivariable model including both year of publication and pork consumption, year of publication remained independently associated with seroprevalence (β = 0.0644, *p* = 0.0080), whereas pork consumption was not independently associated (β = 0.0099, *p* = 0.3341). On the odds scale, each additional year of publication was associated with approximately 6.7% higher odds of reported HEV seroprevalence (OR = 1.07, 95% CI 1.02–1.12). In a sensitivity analysis restricted to studies published after 2010 (k = 14), a univariable meta-regression model including national pork consumption showed a positive but non-significant association with seroprevalence (β = 0.0142, *p* = 0.1325).

Serological assay type was significantly associated with reported HEV seroprevalence in meta-regression (QM (df = 6) = 15.18, *p* = 0.0189). Using Abbott ELISA as the reference category, Mikrogen ELISA, other commercial ELISA assays, and in-house or insufficiently described assays showed higher estimated odds of seropositivity compared with Abbott ELISA. However, these comparisons should be interpreted with caution due to the limited number of studies across several assay categories. These differences likely reflect methodological variation in assay sensitivity, antigen composition, and cut-off definitions rather than true epidemiological differences.

Population type (general population vs. blood donors) was not significantly associated with seroprevalence (β = −0.7915, *p* = 0.1433). In analyses restricted to studies reporting mean participant age (k = 22), mean age was not significantly associated with seroprevalence (β = −0.0328, *p* = 0.4634). Similarly, multilevel meta-regression accounting for within-study clustering of sex-stratified estimates (k = 31 strata from 16 studies) showed no significant association between sex and HEV seroprevalence (β = −0.0158, *p* = 0.8953).

Overall, meta-regression analyses indicated that the year of publication and the serological assay type were significant moderators of reported HEV seroprevalence. In contrast, national pork consumption, population type, mean age, and sex were not significantly associated with seroprevalence. However, substantial residual heterogeneity persisted across all models (e.g., τ^2^ = 1.15; I^2^ = 96.5% in the multivariable model including year of publication and pork consumption), suggesting that these variables explain only a limited proportion of the between-study variability. The remaining heterogeneity likely reflects additional unmeasured epidemiological and methodological factors across studies. Detailed regression coefficients and model diagnostics are presented in Table A4.

### 3.5. Leave-One-Out (LOO) Sensitivity Analysis

**Figure 6:**
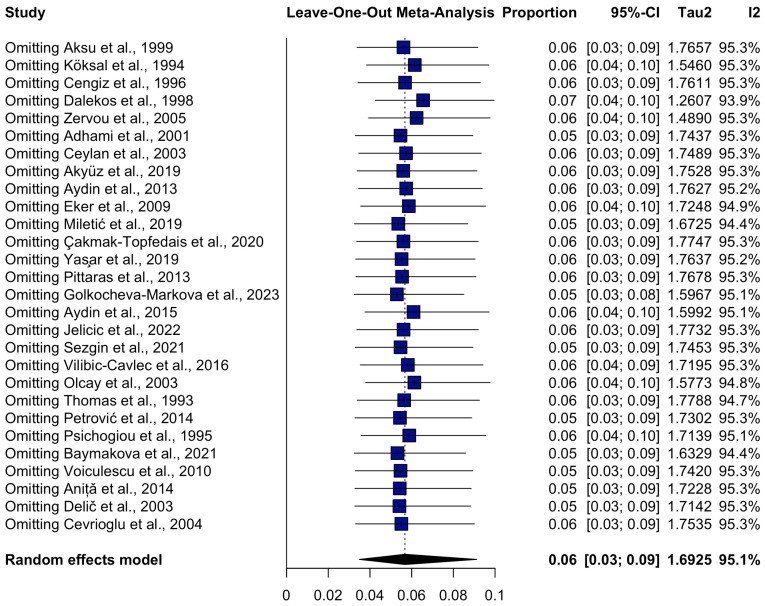
Leave-one-out sensitivity analysis of included studies (PLOGIT–GLMM model). Forest plot showing the pooled anti-HEV seroprevalence after sequentially excluding individual studies. Each row represents the pooled prevalence recalculated after omitting one study. The 95% confidence interval around the pooled estimate is indicated by 95% CI. Tau^2^ represents the between-study variance in the random-effects model. I^2^ (%) quantifies the proportion of total variability attributable to between-study heterogeneity rather than sampling error.

Leave-one-out sensitivity analysis demonstrated high robustness of the pooled HEV seroprevalence estimate. Sequential exclusion of individual studies resulted in minimal changes to the pooled prevalence, which consistently ranged from approximately 5% to 7% and had overlapping confidence intervals. No single study substantially influenced the overall estimate or explained the observed heterogeneity, which remained high across all iterations (Figure 6). These findings indicate that between-study heterogeneity reflects genuine epidemiological and methodological diversity rather than the influence of individual outlier studies.

### 3.6. Publication Bias

**Figure A9:**
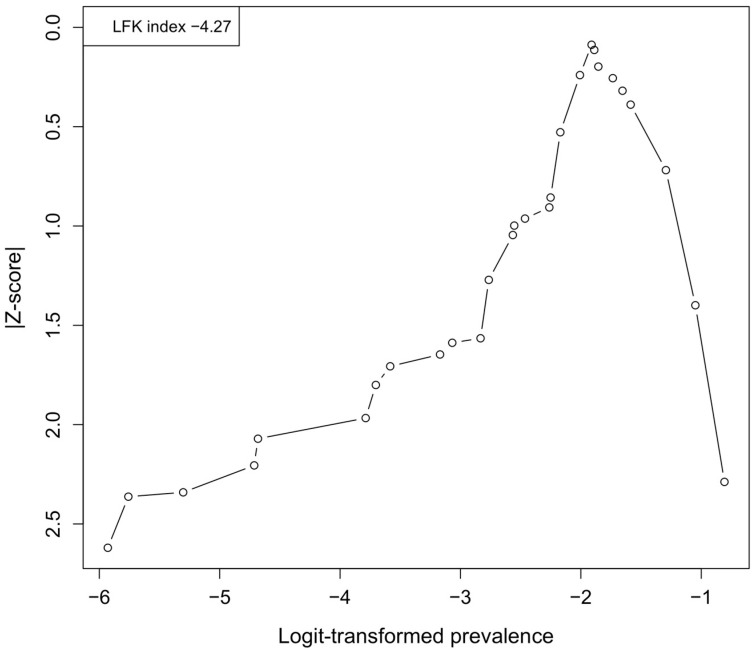
Doi plot for assessment of small-study effects in the included studies. The Luis Furuya–Kanamori (LFK) index was −4.27, indicating major asymmetry.

Small-study effects were assessed using the Doi plot and the Luis Furuya–Kanamori (LFK) index. The Doi plot demonstrated major asymmetry, with an LFK index of −4.27, indicating substantial asymmetry among the included studies (Figure A9). Given the considerable between-study heterogeneity and methodological differences across studies, the observed asymmetry may reflect factors other than publication bias.

## 4. Discussion

Following the initial identification of HEV in animal reservoirs in the early 1990s, a growing body of research demonstrated that this zoonotic infection has a global impact, expanding beyond the traditionally recognized endemic regions. Data on HEV seroprevalence in the Balkan region first emerged from studies conducted in Turkey (1993), Greece (1995), and Bulgaria (1996) [71,89,91]. Despite more than three decades of research, reported HEV estimates remain highly variable across countries and study populations. Furthermore, it should be taken into account that high anti-HEV IgG seroprevalence reflects cumulative past exposure to infection rather than active or clinically apparent disease. Most HEV infections in immunocompetent individuals are asymptomatic or self-limiting and therefore remain clinically unrecognized. Clinically identified cases represent only a small proportion of all HEV infections and occur mainly in vulnerable groups [92].

This systematic review with meta-analysis provides the first comprehensive synthesis of HEV seroprevalence among adults in the general population and blood donors across Balkan countries. A total of 28 eligible studies were identified, with considerable variation in the number of studies per country. Albania contributed the fewest eligible studies, while Turkey contributed the most (*n* = 14). No eligible human seroprevalence studies were identified from Bosnia and Herzegovina, Kosovo, Montenegro, North Macedonia, or Slovenia. Based on all eligible studies, the pooled anti-HEV seroprevalence in the Balkans was estimated at 5.68% (95% CI: 3.48–9.12%). The heterogeneity was substantial (I^2^ = 95.1%) between studies (the pooled HEV seroprevalence ranged from approximately 1% in Greece to over 26% in Bulgaria) (Table 3). These results indicate that zoonotic HEV exposure in the Balkans is widespread but highly variable, influenced by multiple epidemiological factors. These results are consistent with those reported by Wilhelm et al., who observed substantial variability in HEV seroprevalence across non-endemic European countries. In their analysis, seroprevalence ranged from below 5% in several Northern European countries to over 20–30% in countries such as France and Germany, with even higher estimates reported when highly sensitive assays (such as Wantai) were used [51]. Serological evidence for HEV circulation in the Balkans is further supported by molecular analyses confirming HEV-3 transmission in the region [30,93].

### 4.1. Assay Variability, Temporal Trends, and Interpretability of Seroprevalence Estimates

A significant increase in HEV seroprevalence has been reported over the last decade, largely due to improved sensitivity and specificity of the new recombinant assays. A significant association was observed between year of publication and reported HEV seroprevalence (Table 3). Meta-regression results showed that the recent studies tend to report higher seroprevalence estimates (Figure 5). Comparable trends have been documented in other HEV seroepidemiological studies from non-endemic countries and likely reflect improvements in diagnostic tests, increased attention to HEV infection, and broader testing practices, rather than a true increase in infection incidence [49]. The availability of multiple commercial anti-HEV assays, each with varying sensitivity and specificity, further complicates the comparisons of seroprevalence estimates across studies. For example, in a cohort of 1,036 blood donors in Croatia, the overall seroprevalence (IgG/IgM) was 21.5%. However, anti-HEV IgG results varied substantially across assays, ranging from 9.6% with the older recomWell assay to 20.2% with the Dia. Pro assay, while IgM reactivity ranged from 1.5% to 4.4% across assays [67]. The differences in assay performance may substantially affect estimated seroprevalence and complicate comparisons between studies.

The present meta-analysis finds a significant association between assay type and reported seroprevalence, highlighting the influence of diagnostic methodology on HEV epidemiology. However, several assay categories were represented by only a limited number of studies, restricting the robustness of direct comparisons between specific diagnostic platforms. Consequently, differences between assays may reflect methodological variability rather than true epidemiological differences in HEV exposure across populations.

### 4.2. Geographic Variability and Ecological Interpretation

The results from this study demonstrate significant heterogeneity in HEV seroprevalence among Balkan countries (Table 3). However, the comparisons were based on a limited number of studies per country, reduce the precision of the country-specific estimates. Multiple determinants can influence HEV exposure in a studied country, including dietary habits, prevalence in animals, rurality, sanitation, and surveillance capacity. Data from migrant populations suggest that factors beyond pork consumption may influence HEV seroprevalence in the Balkans. For example, a study from the Netherlands found that Turkish migrants, originating from a country with very low pork consumption and minimal pig farming, exhibited a notable HEV seroprevalence (33.4%) [94]. Seropositivity was higher among people who migrated at older ages, indicating likely exposure in their country of origin. These data support the possible role of alternative transmission pathways, including fecal–oral exposure. Additionally, the relatively high seroprevalence observed in these populations may be consistent with the circulation of non-zoonotic HEV genotypes, although molecular confirmation is lacking.

### 4.3. Diet and Pork Consumption: Supportive Signal with Ecological Constraints

Subgroup analyses revealed a higher pooled seroprevalence in countries with high per capita pork consumption (Table 3). This observation is biologically plausible, given the established role of HEV-3 as a food-borne zoonosis associated with undercooked pork and wild boar meat products [95]. Our findings support the hypothesis that dietary exposure to pork products may contribute to regional differences in HEV transmission. At the same time, transmission risk likely depends not only on the quantity of pork consumed but also on food preparation, culinary habits, and contact with animal reservoirs.

However, pork consumption was not independently associated with seroprevalence in meta-regression models (Appendix A, Table A4). As meta-regression evaluates linear associations across studies and is methodologically more robust than simple subgroup comparisons, this discrepancy suggests that the subgroup signal should be interpreted within a broader epidemiological context rather than as an isolated causal relationship.

Moreover, HEV has also been detected in small ruminants, such as goats and sheep, and viral RNA has been identified in raw milk from these species, suggesting additional food-borne exposure pathways [96,97,98]. In several Balkan countries with relatively low pork consumption, goat and sheep meat and dairy products may represent alternative routes of zoonotic exposure.

Environmental transmission of HEV is also a relevant consideration. HEV contamination of irrigation water and fresh produce (leafy greens and berries) has been reported, particularly in areas where agricultural fertilization involves untreated animal manure [99]. HEV RNA was detected in 3.3% and 16.7% of surface water samples in Slovenia and Serbia, respectively [100]. These environmental pathways may contribute to population-level exposure independent of direct meat consumption [53]. Furthermore, bivalve shellfish have been identified as potential vehicles for HEV transmission, as they can bioaccumulate the virus in sewage-impacted coastal waters. Studies from Spain detected HEV RNA in 24.4% of shellfish samples, all of which belonged to swine genotype 3, supporting the hypothesis that shellfish contribute to human exposure [101,102].

The potential role of rat HEV (*Rocahepevirus ratti*) also warrants consideration. Although genetically distinct from HEV-3, rat-associated HEV has been increasingly recognized as capable of infecting humans, suggesting that urban rodent reservoirs may represent an additional source of exposure [28]. Furthermore, HEV-3 RNA has been detected in wild Norway rats (*Rattus norvegicus*) captured near HEV-endemic pig farms, with viral sequences genetically identical to those of circulating swine strains [103]. This evidence further supports the possibility that rodents contribute to environmental maintenance and local dissemination of HEV-3.

Importantly, substantial residual heterogeneity remained across meta-regression models, indicating that the examined moderators explained only part of the between-study variability. In summary, based on the meta-analysis results, our findings suggest that HEV epidemiology in the Balkans is shaped by multiple interacting dietary, environmental, and ecological factors beyond those available for meta-analytic modeling.

### 4.4. Population Characteristics and Reporting Limitations

Pooled seroprevalence did not vary significantly between blood donors and the general population, nor between males and females. The demographic data for the studied populations were incomplete, which limited the interpretation of subgroup findings. In addition, donor-based serosurveys may be biased if specific subpopulations are overrepresented, potentially skewing national estimates and masking subnational variation.

Although HEV is associated with older age, this factor was not statistically significant in our analysis. This likely reflects methodological limitations rather than a true absence of association. Inconsistent reporting on study-level mean age may have obscured age-specific trends and introduced ecological bias. Furthermore, the analysis was restricted to predominantly adult populations, resulting in a more homogeneous study population.

Rural residence is an established risk factor for HEV infection due to closer contact with animal reservoirs, including domestic pigs, wild boar, small ruminants, and rodents. Traditional small-scale pig husbandry and backyard slaughter, which are common in many Balkan rural communities, increase the risk of zoonotic and food-borne transmission [66]. In addition to direct animal contact, rural residents may be exposed through contact with animal manure or water sources contaminated by infected livestock [104]. Populations with regular rural animal contact exhibit substantially higher HEV seroprevalence than general or blood donor cohorts [104]. In the United States, individuals who consume homegrown fruits and vegetables have higher HEV-specific antibody levels [105].

However, only a limited number of studies reported the urban–rural distribution of participants, precluding subgroup analysis by residence. This represents a significant epidemiological gap, as rural populations have been shown to exhibit substantially higher seroprevalence compared to general populations [106]. Incomplete reporting of age structure, sex distribution, sampling frames, and urban–rural composition limited the ability to perform more detailed moderator analyses and likely contributed to residual heterogeneity. Standardized reporting of key demographic and methodological variables would improve comparability between studies and strengthen future evidence syntheses.

## 5. Limitations, Strengths, and Implications for Future Research

There are several limitations to consider when interpreting the results of this meta-analysis. Firstly, subgroup and meta-regression analyses reveal a marked heterogeneity across studies. These data suggest that HEV epidemiology in the Balkans is influenced by other epidemiological and methodological factors that were not included in the present study. Second, the meta-regression was based on study-level (ecological) variables, such as national pork consumption and publication year, precluding individual-level inference and possibly introducing ecological bias. Importantly, national pork consumption was used as a proxy for dietary exposure and did not account for heterogeneity within countries, individual dietary habits, or changes in consumption patterns over time.

Third, variability in serological assay performance, such as differences in antigen composition, sensitivity, and specificity, may have affected the reported seroprevalence. Fourth, demographic and exposure variables, including age structure, urban–rural distribution, and sampling characteristics, were inconsistently reported across studies, limiting more detailed moderator analyses. Finally, geographic representation within the Balkans was uneven, with several countries represented by few or no eligible studies.

This study has several strengths. It is the first comprehensive meta-analysis of HEV seroprevalence in the Balkan region, integrating data from both general populations and blood donors. The use of a generalized linear mixed model (GLMM) facilitated robust pooling across studies with heterogeneous sample sizes and low prevalence rates. Sensitivity analyses, including alternative transformations and leave-one-out procedures, confirmed the stability of the findings.

## 6. Conclusions

HEV seroprevalence among adults in the studied region is heterogeneous and seems to be strongly influenced by methodological factors, including the type of serological assay and the study period. Harmonized surveillance and standardized diagnostic approaches within a One Health framework are essential for accurately characterizing HEV burden and developing prevention strategies in the Balkans.

## Figures and Tables

**Table 1:** Characteristics of the studies included in the meta-analysis of HEV seroprevalence in the Balkan region, including country, population type, sample size, age distribution, sex distribution, and serological assay used (manufacturer and antibody target). (*n*) means the number of individuals.

Study	Population Type	Population (*n*)	Age (Range; Mean)	Male (*n*; %)	Test (Manufacturer; Target)	Ref.
**Albania**						
Adhami et al., 2001	General population ^α^	388	≥20 y; n/a	n/a	Abbott, Germany; IgG & IgM	[63]
**Bulgaria**						
Baymakova et al., 2021	Blood donors	555	≥18; 37.2	479; 86%	Mikrogen, Germany; IgG	[64]
Golkocheva-Markova et al., 2023	Blood donors	94	19–60; 36.5 ^β^	77; 82%	Euroimmun, Germany, or DiaPro, Italy; IgG or IgM	[65]
**Croatia**						
Jelicic et al., 2022	General population ^α^	126	33–62; 47.7 ^β^	n/a	Euroimmun, Germany; IgG	[66]
Miletić et al., 2019	Blood donors	1036	18–69; 44.7 ^β^	913; 88%	Dia. Pro, Italy; IgG & IgM	[67]
Vilibic-Cavlec et al., 2016	General population	37	≥18; n/a	n/a	Euroimmun, Germany; IgG or IgM	[68]
**Greece**						
Dalekos et al., 1998	Blood donors	3016	18–60; 43 ^β^	2473; 82%	Abbott, Germany; IgG	[69]
Pittaras et al., 2013	Blood donors	265	19–61; 39.6	216; 81.5%	EIAgen, Italy; IgG	[70]
Psichogiou et al., 1995	General population	316	16–87; 33.8	266; 84%	in-house ELISA; IgG	[71]
Zervou et al., 2005	General population	158	40–79; 59.1 ^γ^	135; 85%	Abbott, Germany; IgG	[72]
**Romania**						
Anițǎ et al., 2014	General population	137	18–90; n/a	40; 51% ^δ^	MP Biomedicals; IgG	[73]
Voiculescu et al., 2010	General population	137 ^ε^	≥18; 32.28 ^γ^	21; 15%	Mikrogen, Germany; IgG	[74]
**Serbia**						
Delič et al., 2003	Blood donors	206	≥18 ^ζ^	n/a	n/a; IgG	[75]
Petrović et al., 2014	Blood donors	200	19–65; 39.3	158; 79%	in-house ELISA; IgG	[76]
**Turkey**						
Aksu et al., 1999	General population	51	15–65; 35	29; 57%	Abbott Diagnostics, USA; IgG & IgM	[77]
Akyüz et al., 2019	General population	21	≥18 ^ζ^; 37.5	12; 57%	Dia. Pro, Italy; IgG	[78]
Aydin et al., 2013	Blood donors	248	≥18; 39.6 ^γ^	137; 55%	Dia. Pro, Italy; IgG	[79]
Aydin et al., 2015	Blood donors	327	19–59; 31.1	295; 92%	Euroimmun, Germany; IgG	[80]
Çakmak-Topfedais, 2020	General population	180	17–73; 36.6	69; 38%	Dia. Pro, Italy; IgG or IgM	[81]
Cengiz et al., 1996	General population	54	22–74; 46.5	16; 30%	Abbott, Germany; IgG	[82]
Cevrioglu et al., 2004	General population	76	19–42; 27.5	0; 0%	Virotech GmbH, Germany; IgG or IgM	[83]
Ceylan et al., 2003	General population	45	≥15; 28.5	37; 82%	Bioser, Italy; IgG	[84]
Eker et al., 2009	General population	582	≥15; 40.9	273; 47%	Dia. Pro, Italy; IgG	[85]
Köksal et al., 1994	General population	100	18–41; 24.7	63; 63%	Abbott Laboratories, USA; IgG	[86]
Olcay et al., 2003	General population	787	≥15; n/a	n/a	Genelabs Diagnostics, Switzerland; IgG	[87]
Sezgin et al., 2021	Blood donors	900	≥18 ^ζ^; 35.2	889; 99%	Euroimmun, Germany; IgG	[88]
Thomas et al., 1993	General population	1350	≥15; n/a	672; 50%	Genelabs Technologies, USA; IgG & IgM	[89]
Yaşar et al., 2019	Blood donors	2011	18–65; 35.8	1870; 93%	Dia.Pro, Italy and Wantai, China; IgG & IgM ^η^	[90]

^α^—Author-defined general population; ^β^—mean age estimated from the reported median; ^γ^—mean age was recalculated when studies reported separate subgroup means (e.g., by sex or occupation) by deriving a combined mean weighted by subgroup sample sizes; ^δ^—totals derived from a subset of the population of interest (the 2012 cohort). Sex-stratified seroprevalence data were available only for the 2012 cohort; ^ε^—four participants were not tested for HEV. Sex-stratified seroprevalence data were available only for the healthcare professionals cohort; ^ζ^—adult population (minimum age not reported); ^η^—seropositivity was defined as concordant (double-positive) results across two assays, as reported by the original study.

**Table 2:** National pork consumption (kg/person/year, 2023) in Balkan countries included in this review. Estimates were derived from FAO Food Balance Sheets (FAOSTAT).

Country	Pork (kg/person/year)	Consumption Category
Albania	7.51	Low
Bulgaria	32.8	Moderate
Croatia	61.06	High
Greece	27.82	Moderate
Romania	39.28	High
Serbia	50.59	High
Turkey	~0	Very low

**Table 3:** Pooled HEV seroprevalence across key subgroups in the Balkans was estimated using a random-effects model (PLOGIT–GLMM). No. of studies indicates the number of independent datasets included in each subgroup. Events/Total represents the cumulative number of seropositive individuals and the total sample size within each subgroup. Pooled prevalence (%) shows the summary seroprevalence estimate with corresponding 95% confidence interval (95% CI), reflecting the precision of the pooled estimate. I^2^ (%) quantifies the proportion of total variability attributable to between-study heterogeneity rather than chance, with higher values indicating greater heterogeneity. *p*-values correspond to tests for subgroup differences (χ^2^ test) within each variable.

Variable	No of Studies	Events/Total	Pooled Prevalence (%) [95% CI]	I^2^ (%)	*p*-Value
Pork consumption					
Very low/Low	15	520/7120	4.71 [2.85–7.68]	89.8	
Moderate	6	213/4404	3.33 [0.53–18.33]	97.9	
High	7	338/1875	13.99 [10.28–18.74]	75.9	0.0005 *
Assay					
Abbott ELISA	6	66/3767	1.25 [0.19–7.57]	95.5	
Dia.Pro ELISA	5	262/2067	6.7 [3.08–13.96]	96.4	
Euroimmun ELISA	4	129/1390	4.19 [1.36–12.16]	88.9	
Genelabs ELISA	2	87/2137	2.39 [0.61–8.91]	95.9	
Mikogen ELISA	2	162/688	19.86 [12.36–30.35]	88.7	
Other commercial ELISA	6	293/2628	12.75 [8.24–19.21]	87.3	
In-house ELISA or unclear	3	72/722	8.71 [3.02–22.64]	92.8	0.0011 *
Population type					
General population	20	314/7857	4.46 [2.4–8.14]	93.3	
Blood donors	8	757/5542	9.7 [5.06–17.79]	96	0.0854
Year of publication					
<2000	6	102/4887	1.89 [0.6–5.84]	93.5	
2000–2010	8	136/2375	4.56 [1.73–11.51]	92.5	
>2010	14	833/6137	10 [6.32–15.49]	93.8	0.0155 *
Sex					
Male	15	567/5381	5.96 [2.88–11.91]	92.5	
Female	16	135/1836	7.97 [5.3–11.8]	76.5	0.4843
Country					
Albania	1	51/388	13.14 [9.95–16.92]	-	
Bulgaria	2	173/649	26.66 [23.4–30.19]	0	
Croatia	3	233/1199	9.41 [3.46–23.13]	88.7	
Greece	4	40/3755	1.01 [0.16–6.13]	96.3	
Romania	2	40/270	14.81 [11.06–19.57]	0	
Serbia	2	65/406	16.01 [12.76–19.91]	0	
Turkey	14	469/6732	4.31 [2.57–7.14]	89.8	<0.0001 *

*—statistically significant.

## Data Availability

No new data are available.

## Supplementary Materials

The following supporting information can be downloaded at: https://www.mdpi.com/article/10.3390/v18070736/s1, Table S1: PRISMA checklist.

## Appendix A

**Table A1:** Complete search strategies used in PubMed, Scopus, and Web of Science for the identification of Studies on HEV seroprevalence in Balkan countries.

Database	Search Strategy
Pubmed	(Balkan* OR “Balkan Peninsula”[MeSH Terms] OR Albania OR “Bosnia and Herzegovina” OR Bulgaria OR Croatia OR Greece OR Kosovo OR Montenegro OR “North Macedonia” OR Romania OR Serbia OR Slovenia OR Turkey OR Turkiye OR Türkiye) AND (“Hepatitis E”[MeSH Terms] OR “Hepatitis E” OR HEV)
Scopus	TITLE-ABS-KEY((Albania OR “Bosnia and Herzegovina” OR Bulgaria OR Croatia OR Greece OR Kosovo OR Montenegro OR “North Macedonia” OR Romania OR Serbia OR Slovenia OR Turkey OR Turkiye OR Türkiye OR Balkan*) AND (“Hepatitis E” OR HEV))
WoS	TS = ((Albania OR “Bosnia and Herzegovina” OR Bulgaria OR Croatia OR Greece OR Kosovo OR Montenegro OR “North Macedonia” OR Romania OR Serbia OR Slovenia OR Turkey OR Turkiye OR Türkiye OR Balkan*) AND (“Hepatitis E” OR HEV))

**Table A2:** Risk of bias assessment of studies included in the meta-analysis. Methodological quality of the included studies was evaluated using the Joanna Briggs Institute (JBI) Critical Appraisal Checklist for Studies Reporting Prevalence Data. The checklist consists of nine domains assessing the appropriateness of the sample frame (Q1), sampling method (Q2), adequacy of sample size (Q3), description of study subjects and setting (Q4), coverage of the identified sample in the analysis (Q5), validity of methods used to identify the condition (Q6), reliability and consistency of condition measurement across participants (Q7), appropriateness of statistical analysis (Q8), and adequacy of response rate or management of low response rate (Q9). Each item was rated as Yes (Y), No (N), or Unclear (U). Overall risk of bias was categorized based on the number of “Yes” responses: low risk (7–9), moderate risk (4–6), and high risk (0–3).

	Study	Q1	Q2	Q3	Q4	Q5	Q6	Q7	Q8	Q9	Overall RoB	References
1	Adhami et al., 2001	Y	U	Y	Y	Y	Y	Y	Y	U	L	[63]
2	Aksu et al., 1999	U	N	N	Y	Y	Y	Y	Y	U	M	[77]
3	Akyüz et al., 2019	U	U	N	Y	Y	Y	Y	Y	U	M	[78]
4	Aniță et al., 2014	U	N	Y	Y	Y	Y	Y	Y	U	M	[73]
5	Aydin et al., 2013	Y	Y	Y	Y	Y	Y	Y	Y	Y	L	[79]
6	Aydin et al., 2015	Y	Y	Y	Y	Y	Y	Y	Y	Y	L	[80]
7	Baymakova et al., 2021	Y	Y	Y	Y	Y	Y	Y	Y	Y	L	[64]
8	Çakmak-Topfedais, 2020	N	N	Y	Y	Y	Y	Y	Y	U	M	[81]
9	Cengiz et al., 1996	U	U	N	Y	Y	Y	Y	Y	U	M	[82]
10	Cevrioglu et al., 2004	Y	U	N	Y	Y	Y	Y	Y	U	M	[83]
11	Ceylan et al., 2003	Y	N	N	Y	Y	Y	Y	Y	U	M	[84]
12	Dalekos et al., 1998	Y	Y	Y	Y	Y	Y	Y	Y	Y	L	[69]
13	Delić et al., 2003	Y	U	Y	U	Y	U	U	Y	U	M	[75]
14	Eker et al., 2009	Y	Y	Y	Y	Y	Y	Y	Y	Y	L	[85]
15	Golkocheva-Markova et al., 2023	Y	U	N	Y	Y	Y	U	Y	U	M	[65]
16	Jelicic et al., 2022	Y	U	Y	Y	Y	Y	Y	Y	U	L	[66]
17	Köksal et al., 1994	Y	N	Y	Y	Y	Y	Y	Y	U	L	[86]
18	Miletic et al., 2019	Y	Y	Y	Y	Y	Y	Y	Y	Y	L	[67]
19	Olcay et al., 2003	Y	Y	Y	Y	Y	Y	Y	Y	Y	L	[87]
20	Petrović et al., 2014	Y	Y	Y	Y	Y	Y	Y	Y	Y	L	[76]
21	Pittaras et al., 2013	Y	Y	Y	Y	Y	Y	Y	Y	Y	L	[70]
22	Psichogiou et al., 1995	Y	N	Y	Y	Y	N	Y	Y	U	M	[71]
23	Sezgin et al., 2021	Y	Y	Y	Y	Y	Y	Y	Y	Y	L	[88]
24	Thomas et al., 1993	Y	Y	Y	Y	Y	Y	Y	Y	Y	L	[89]
25	Vilibic-Cavlek et al., 2016	N	U	N	Y	Y	Y	Y	Y	U	M	[68]
26	Voiculescu et al., 2010	N	N	Y	Y	Y	Y	Y	Y	U	M	[74]
27	Yaşar et al., 2019	Y	Y	Y	Y	Y	Y	Y	Y	Y	L	[90]
28	Zervou et al., 2005	Y	N	Y	Y	Y	Y	Y	Y	U	L	[72]

**Table A3:** Studies excluded from the meta-analysis after full-text review, with corresponding reasons for exclusion based on predefined eligibility criteria.

Study	Country	Exclusion Reason	Reference
Aydin et al., 2016	Turkey	Possible overlapping study population with Aydin et al., 2013 [79].	[107]
Bayram et al., 2007	Turkey	Hospital-based control group; participants were recruited from a clinical setting and did not meet the predefined general population criteria.	[108]
Cesur et al., 2002	Turkey	Hospital outpatient clinic-based sampling; participants did not meet predefined general population or blood donor inclusion criteria.	[109]
Gorski et al., 2023	Croatia	Reported HEV RNA prevalence only; no seroprevalence (IgG/IgM) data available.	[110]
Mladenova-Dimitrova et al., 2020	Bulgaria	Mixed clinical and prophylactic cohort; population did not meet predefined general population criteria.	[111]
Psichogiou et al., 1996	Greece	Overlapping cohort with Psichogiou et al., 1995 [71]; duplicate population.	[112]
Psichogiou et al., 1996	Greece	Overlapping cohort with Psichogiou et al., 1995 [71]; duplicate population.	[113]
Teoharov et al., 2014	Bulgaria	Insufficient extractable data for the predefined adult population.	[114]
Thomas et al., 1994	Turkey	Overlapping cohort with Thomas et al., 1993 [89]; less detailed HEV-specific data.	[115]
Yaşar et al., 2025	Turkey	Overlapping cohort with Sezgin et al., 2021 [88].	[116]

**Table A4:** Mixed-effects meta-regression models exploring study-level determinants of HEV seroprevalence. Models include univariable, multivariable (year of publication and pork consumption), multilevel sex, and restricted post-2010 sensitivity analyses. k indicates the number of studies included in each model. β (logit) represents the regression coefficient estimated on the logit-transformed prevalence scale, with SE denoting the standard error and 95% CI the corresponding confidence interval. QM tests the overall significance of moderator(s). τ^2^ and I^2^ describe the amount and proportion of residual between-study heterogeneity, respectively. In the multilevel sex model, σ^2^ denotes the estimated between-study variance and QE tests residual heterogeneity.

Model/Moderator	k	β (logit)	SE	95% CI	*p*-Value	QM (df)	QM p	Residual τ^2^	Residual I^2^
Univariable models									
Year of publication	28	0.0716	0.0237	0.0251 to 0.1181	0.0025 *	9.11 (1)	0.0025 *	1.2158	97.14%
Pork consumption (kg/person/year)	28	0.0180	0.0110	−0.0035 to 0.0394	0.1012	2.69 (1)	0.1012	1.4948	97.46%
Population type (general population vs. donors)	28	−0.7915	0.5408	−1.8516 to 0.2685	0.1433	2.14 (1)	0.1433	1.5593	97.77%
Mean age	22	−0.0328	0.0448	−0.1206 to 0.0549	0.4634	0.54 (1)	0.4634	1.7982	97.78%
Restricted sensitivity analysis (post-2010; univariable)									
Pork consumption	14	0.0142	0.0094	−0.0043 to 0.0326	0.1325	2.26 (1)	0.1325	0.6344	95.62%
Serological assay type (reference: Abbott ELISA)									
Overall	28	-	-	-		15.18 (6)	0.0189 *	1.0374	93.82%
Dia.Pro ELISA	28	1.3611	0.6962	−0.0033 to 2.7256	0.0506	-	-	-	-
Euroimmun ELISA	28	0.8777	0.7525	−0.5973 to 2.3526	0.2435	-	-	-	-
Genelabs ELISA	28	0.2874	0.8947	−1.4661 to 2.0410	0.7480	-	-	-	-
In-house/unclear ELISA	28	1.6485	0.7843	0.1113 to 3.1856	0.0356 *	-	-	-	-
Mikrogen ELISA	28	2.5494	0.8859	0.8131 to 4.2857	0.0040 *	-	-	-	-
Other commercial ELISA	28	2.0249	0.6610	0.7294 to 3.3204	0.0022 *	-	-	-	-
Multivariable model (Year + Pork consumption)									
Overall	28	-	-	-	-	10.47 (2)	0.0053 *	1.1490	96.46%
Year of publication	28	0.0644	0.0243	0.0168 to 0.1119	0.0080 *	-	-	-	-
Pork consumption	28	0.0099	0.0102	−0.0101 to 0.0299	0.3341	-	-	-	-
Multilevel model (Sex; 31 strata from 16 studies)		*σ* ^2^				QE p			
Female vs. male	31	−0.0158	0.1197	−0.2504 to 0.2189	0.8953	0.02 (1)	0.8953	0.8177	<0.0001 *

*—statistically significant (*p* < 0.05).

## References

[1] Khuroo M.S., Khuroo M.S., Khuroo N.S. (2016). Hepatitis E: Discovery, Global Impact, Control and Cure. World J. Gastroenterol..

[2] Harrison L., DiCaprio E. (2018). Hepatitis E Virus: An Emerging Foodborne Pathogen. Front. Sustain. Food Syst..

[3] Mazalovska M., Varadinov N., Koynarski T., Minkov I., Teoharov P., Lomonossoff G.P., Zahmanova G. (2017). Detection of Serum Antibodies to Hepatitis E Virus Based on HEV Genotype 3 ORF2 Capsid Protein Expressed in Nicotiana Benthamiana. Ann. Lab. Med..

[4] Meng X.J., Purcell R.H., Halbur P.G., Lehman J.R., Webb D.M., Tsareva T.S., Haynes J.S., Thacker B.J., Emerson S.U. (1997). A Novel Virus in Swine Is Closely Related to the Human Hepatitis E Virus. Proc. Natl. Acad. Sci. USA.

[5] Augustyniak A., Pomorska-Mól M. (2023). An Update in Knowledge of Pigs as the Source of Zoonotic Pathogens. Animals.

[6] Belei O., Ancusa O., Mara A., Olariu L., Amaricai E., Folescu R., Zamfir C.L., Gurgus D., Motoc A.G., Stânga L.C. (2021). Current Paradigm of Hepatitis E Virus Among Pediatric and Adult Patients. Front. Pediatr..

[7] Khuroo M.S., Kamili S. (2003). Aetiology, Clinical Course and Outcome of Sporadic Acute Viral Hepatitis in Pregnancy. J. Viral Hepat..

[8] Liu H., Ma Y. (2020). Hepatitis E Virus-Associated Guillain-Barre Syndrome: Revision of the Literature. Brain Behav..

[9] Purdy M.A., Drexler J.F., Meng X.-J., Norder H., Okamoto H., Van der Poel W.H.M., Reuter G., de Souza W.M., Ulrich R.G., Smith D.B. (2022). ICTV Virus Taxonomy Profile: Hepeviridae 2022. J. Gen. Virol..

[10] Prpić J., Baymakova M. (2023). Hepatitis E Virus (HEV) Infection among Humans and Animals: Epidemiology, Clinical Characteristics, Treatment, and Prevention. Pathogens.

[11] Ryll R., Bernstein S., Heuser E., Schlegel M., Dremsek P., Zumpe M., Wolf S., Pépin M., Bajomi D., Müller G. (2017). Detection of Rat Hepatitis E Virus in Wild Norway Rats (Rattus Norvegicus) and Black Rats (Rattus Rattus) from 11 European Countries. Vet. Microbiol..

[12] Smith D.B., Izopet J., Nicot F., Simmonds P., Jameel S., Meng X.-J., Norder H., Okamoto H., van der Poel W.H.M., Reuter G. (2020). Update: Proposed Reference Sequences for Subtypes of Hepatitis E Virus (Species Orthohepevirus A). J. Gen. Virol..

[13] Nelson K.E., Labrique A.B., Kmush B.L. (2019). Epidemiology of Genotype 1 and 2 Hepatitis E Virus Infections. Cold Spring Harb. Perspect. Med..

[14] Doceul V., Bagdassarian E., Demange A., Pavio N. (2016). Zoonotic Hepatitis E Virus: Classification, Animal Reservoirs and Transmission Routes. Viruses.

[15] Zahmanova G., Takova K., Lukov G.L., Andonov A. (2024). Hepatitis E Virus in Domestic Ruminants and Virus Excretion in Milk-A Potential Source of Zoonotic HEV Infection. Viruses.

[16] Takova K., Koynarski T., Minkov I., Ivanova Z., Toneva V., Zahmanova G. (2020). Increasing Hepatitis E Virus Seroprevalence in Domestic Pigs and Wild Boar in Bulgaria. Animals.

[17] Aslan A.T., Balaban H.Y. (2020). Hepatitis E Virus: Epidemiology, Diagnosis, Clinical Manifestations, and Treatment. World J. Gastroenterol..

[18] Hakze-van der Honing R.W., van Coillie E., Antonis A.F.G., van der Poel W.H.M. (2011). First Isolation of Hepatitis E Virus Genotype 4 in Europe through Swine Surveillance in the Netherlands and Belgium. PLoS ONE.

[19] Tessé S., Lioure B., Fornecker L., Wendling M.-J., Stoll-Keller F., Bigaillon C., Nicand E. (2012). Circulation of Genotype 4 Hepatitis E Virus in Europe: First Autochthonous Hepatitis E Infection in France. J. Clin. Virol..

[20] Lee G.-H., Tan B.-H., Teo E.C.-Y., Lim S.-G., Dan Y.-Y., Wee A., Aw P.P.K., Zhu Y., Hibberd M.L., Tan C.-K. (2016). Chronic Infection With Camelid Hepatitis E Virus in a Liver Transplant Recipient Who Regularly Consumes Camel Meat and Milk. Gastroenterology.

[21] Santos-Silva S., Hemnani M., Lopez-Lopez P., Gonçalves H.M.R., Rivero-Juarez A., Van der Poel W.H.M., Nascimento M.S.J., Mesquita J.R. (2023). A Systematic Review of Hepatitis E Virus Detection in Camels. Vet. Sci..

[22] Pires H., Cardoso L., Lopes A.P., Fontes M.d.C., Santos-Silva S., Matos M., Pintado C., Figueira L., Matos A.C., Mesquita J.R. (2023). Prevalence and Risk Factors for Hepatitis E Virus in Wild Boar and Red Deer in Portugal. Microorganisms.

[23] Castagna F., Liguori G., Lombardi R., Bava R., Costagliola A., Giordano A., Quintiliani M., Giacomini D., Albergo F., Gigliotti A. (2024). Hepatitis E and Potential Public Health Implications from a One-Health Perspective: Special Focus on the European Wild Boar (Sus Scrofa). Pathogens.

[24] Sridhar S., Yip C.C.Y., Wu S., Cai J., Zhang A.J.-X., Leung K.-H., Chung T.W.H., Chan J.F.W., Chan W.-M., Teng J.L.L. (2018). Rat Hepatitis E Virus as Cause of Persistent Hepatitis after Liver Transplant. Emerg. Infect. Dis..

[25] Soriano V., Moreno-Torres V., Vázquez E., Álvarez-Domínguez C., Oteo J.A., Mendoza C. (2026). de Spreading of the New Rat Hepatitis E Virus (rHEV) into Humans. NeuroImmune Pharmacol. Ther..

[26] Andonov A., Robbins M., Borlang J., Cao J., Hatchette T., Stueck A., Deschambault Y., Murnaghan K., Varga J., Johnston L. (2019). Rat Hepatitis E Virus Linked to Severe Acute Hepatitis in an Immunocompetent Patient. J. Infect. Dis..

[27] Rivero-Juarez A., Johne R., Sridhar S. (2026). Rocahepevirus Ratti: Molecular Evolution, Zoonotic Potential and Public Health Impact. Nat. Commun..

[28] Santos-Silva S., Gonçalves H.M.R., Van der Poel W.H.M., Nascimento M.S.J., Mesquita J.R. (2025). Rat Hepatitis E Virus (Rocahepevirus Ratti): A Systematic Review of Its Presence in Water, Food-Related Matrices, and Potential Risks to Human Health. Foods.

[29] Santos-Silva S., Lois M., Machado A., Bordalo A., Cruz A.V.S., Gonçalves H.M.R., Van der Poel W.H.M., Nascimento M.S.J., Rivero-Juarez A., Romalde J.L. (2025). Environmental Surveillance of Hepatitis E Virus and Rat Hepatitis E Virus in Portugal and Spain, 2020–2022. J. Med. Virol..

[30] De Sabato L., Ianiro G., Alborali G.L., Kroneman A., Grierson S.S., Krumova-Valcheva G.L., Hakze-van der Honing R.W., Johne R., Kolackova I., Kozyra I. (2023). Molecular Characterization and Phylogenetic Analysis of Hepatitis E Virus (HEV) Strains from Pigs Farmed in Eight European Countries between 2020 and 2022. Transbound. Emerg. Dis..

[31] Dencs Á., Hettmann A., Zsichla L., Müller V., Dömötör A., Barna-Lázár Á., Barcsay E., Takács M. (2025). Molecular Epidemiology of Hepatitis E Virus in Hungary (2018–2025): Emergence of Rare Subtypes and First Detection of HEV-4 in Central Europe. Viruses.

[32] Takova K., Koynarski T., Minkov G., Toneva V., Mardanova E., Ravin N., Lukov G.L., Zahmanova G. (2021). Development and Optimization of an Enzyme Immunoassay to Detect Serum Antibodies against the Hepatitis E Virus in Pigs, Using Plant-Derived ORF2 Recombinant Protein. Vaccines.

[33] Kaufmann A., Kenfak-Foguena A., André C., Canellini G., Bürgisser P., Moradpour D., Darling K.E.A., Cavassini M. (2011). Hepatitis E Virus Seroprevalence among Blood Donors in Southwest Switzerland. PLoS ONE.

[34] Mansuy J.-M., Bendall R., Legrand-Abravanel F., Sauné K., Miédouge M., Ellis V., Rech H., Destruel F., Kamar N., Dalton H.R. (2011). Hepatitis E Virus Antibodies in Blood Donors, France. Emerg. Infect. Dis..

[35] Grabarczyk P., Sulkowska E., Gdowska J., Kopacz A., Liszewski G., Kubicka-Russel D., Baylis S.A., Corman V.M., Noceń E., Piotrowski D. (2018). Molecular and Serological Infection Marker Screening in Blood Donors Indicates High Endemicity of Hepatitis E Virus in Poland. Transfusion.

[36] Cleland A., Smith L., Crossan C., Blatchford O., Dalton H.R., Scobie L., Petrik J. (2013). Hepatitis E Virus in Scottish Blood Donors. Vox Sang..

[37] Wenzel J.J., Preiss J., Schemmerer M., Huber B., Jilg W. (2013). Test Performance Characteristics of Anti-HEV IgG Assays Strongly Influence Hepatitis E Seroprevalence Estimates. J. Infect. Dis..

[38] Kuniholm M.H., Purcell R.H., McQuillan G.M., Engle R.E., Wasley A., Nelson K.E. (2009). Epidemiology of Hepatitis E Virus in the United States: Results from the Third National Health and Nutrition Examination Survey, 1988-1994. J. Infect. Dis..

[39] Bi H., Yang R., Wu C., Xia J. (2020). Hepatitis E Virus and Blood Transfusion Safety. Epidemiol. Infect..

[40] EDQM Publishes 22nd Edition of the Blood Guide—European Directorate for the Quality of Medicines & HealthCare—EDQM. https://www.edqm.eu/en/-/edqm-publishes-22nd-edition-of-the-blood-guide.

[41] Azam B., Marti M., Goel A., Aggarwal R. (2025). Immunogenicity, Efficacy, and Effectiveness of Two-Dose and Shorter Schedules of Hepatitis E Vaccine: A Systematic Review. Vaccines.

[42] Li T.-C., Suzaki Y., Ami Y., Dhole T.N., Miyamura T., Takeda N. (2004). Protection of Cynomolgus Monkeys against HEV Infection by Oral Administration of Recombinant Hepatitis E Virus-like Particles. Vaccine.

[43] Mardanova E.S., Takova K.H., Toneva V.T., Zahmanova G.G., Tsybalova L.M., Ravin N.V. (2020). A Plant-Based Transient Expression System for the Rapid Production of Highly Immunogenic Hepatitis E Virus-like Particles. Biotechnol. Lett..

[44] Huang W., Zhang H., Harrison T.J., Lang S., Huang G., Wang Y. (2008). Cross-Protection of Hepatitis E Virus Genotypes 1 and 4 in Rhesus Macaques. J. Med. Virol..

[45] Zahmanova G.G., Mazalovska M., Takova K.H., Toneva V.T., Minkov I.N., Mardanova E.S., Ravin N.V., Lomonossoff G.P. (2020). Rapid High-Yield Transient Expression of Swine Hepatitis E ORF2 Capsid Proteins in Nicotiana Benthamiana Plants and Production of Chimeric Hepatitis E Virus-Like Particles Bearing the M2e Influenza Epitope. Plants.

[46] Sanford B.J., Opriessnig T., Kenney S.P., Dryman B.A., Córdoba L., Meng X.-J. (2012). Assessment of the Cross-Protective Capability of Recombinant Capsid Proteins Derived from Pig, Rat, and Avian Hepatitis E Viruses (HEV) against Challenge with a Genotype 3 HEV in Pigs. Vaccine.

[47] Nesbitt R.C., Kinya Asilaza V., Alvarez C., Gitahi P., Nkemenang P., Duncker J., Haile M., Gakima P., Wamala J.F., Loro F.B. (2025). The Effectiveness of Two Doses of Recombinant Hepatitis E Vaccine in Response to an Outbreak in Bentiu, South Sudan: A Case-Control and Bias Indicator Study. Lancet Infect. Dis..

[48] Aspinall E.J., Couturier E., Faber M., Said B., Ijaz S., Tavoschi L., Takkinen J., Adlhoch C. (2017). The Country Experts Hepatitis E Virus Infection in Europe: Surveillance and Descriptive Epidemiology of Confirmed Cases, 2005 to 2015. Euro. Surveill..

[49] Hartl J., Otto B., Madden R.G., Webb G., Woolson K.L., Kriston L., Vettorazzi E., Lohse A.W., Dalton H.R., Pischke S. (2016). Hepatitis E Seroprevalence in Europe: A Meta-Analysis. Viruses.

[50] Li P., Liu J., Li Y., Su J., Ma Z., Bramer W.M., Cao W., de Man R.A., Peppelenbosch M.P., Pan Q. (2020). The Global Epidemiology of Hepatitis E Virus Infection: A Systematic Review and Meta-Analysis. Liver Int..

[51] Wilhelm B., Waddell L., Greig J., Young I. (2019). Systematic Review and Meta-Analysis of the Seroprevalence of Hepatitis E Virus in the General Population across Non-Endemic Countries. PLoS ONE.

[52] Pavio N., Kooh P., Cadavez V., Gonzales-Barron U., Thébault A. (2021). Risk Factors for Sporadic Hepatitis E Infection: A Systematic Review and Meta-Analysis. Microb. Risk Anal..

[53] Mrzljak A., Dinjar-Kujundzic P., Jemersic L., Prpic J., Barbic L., Savic V., Stevanovic V., Vilibic-Cavlek T. (2019). Epidemiology of Hepatitis E in South-East Europe in the “One Health” Concept. World J. Gastroenterol..

[54] Mrzljak A., Jemersic L., Savic V., Balen I., Ilic M., Jurekovic Z., Pavicic-Saric J., Mikulic D., Vilibic-Cavlek T. (2021). Hepatitis E Virus in Croatia in the “One-Health” Context. Pathogens.

[55] Fernández Villalobos N.V., Kessel B., Rodiah I., Ott J.J., Lange B., Krause G. (2022). Seroprevalence of Hepatitis E Virus Infection in the Americas: Estimates from a Systematic Review and Meta-Analysis. PLoS ONE.

[56] Mirzaev U.K., Ouoba S., Ko K., Phyo Z., Chhoung C., Ataa A.G., Sugiyama A., Akita T., Tanaka J. (2024). Systematic Review and Meta-Analysis of Hepatitis E Seroprevalence in Southeast Asia: A Comprehensive Assessment of Epidemiological Patterns. BMC Infect. Dis..

[57] Food and Agriculture Organization of the United Nations (2020). Regional Overview of Food Security and Nutrition Nutrition in Europe, Rome.

[58] Page M.J., McKenzie J.E., Bossuyt P.M., Boutron I., Hoffmann T.C., Mulrow C.D., Shamseer L., Tetzlaff J.M., Akl E.A., Brennan S.E. (2021). The PRISMA 2020 statement: An updated guideline for reporting systematic reviews. BMJ.

[59] Munn Z., Barker T.H., Moola S., Tufanaru C., Stern C., McArthur A., Stephenson M., Aromataris E. (2020). Methodological Quality of Case Series Studies: An Introduction to the JBI Critical Appraisal Tool. JBI Evid. Synth..

[60] Wan X., Wang W., Liu J., Tong T. (2014). Estimating the Sample Mean and Standard Deviation from the Sample Size, Median, Range and/or Interquartile Range. BMC Med. Res. Methodol..

[61] Luo D., Wan X., Liu J., Tong T. (2018). Optimally Estimating the Sample Mean from the Sample Size, Median, Mid-Range, and/or Mid-Quartile Range. Stat. Methods Med. Res..

[62] Hunter J.P., Saratzis A., Sutton A.J., Boucher R.H., Sayers R.D., Bown M.J. (2014). In Meta-Analyses of Proportion Studies, Funnel Plots Were Found to Be an Inaccurate Method of Assessing Publication Bias. J. Clin. Epidemiol..

[63] Adhami J.E., Angoni R. (2001). [Hepatitis E virus infection in Albania]. Sante.

[64] Baymakova M., Terzieva K., Popov R., Grancharova E., Kundurzhiev T., Pepovich R., Tsachev I. (2021). Seroprevalence of Hepatitis E Virus Infection among Blood Donors in Bulgaria. Viruses.

[65] Golkocheva-Markova E., Ismailova C., Kevorkyan A., Raycheva R., Zhelyazkova S., Kotsev S., Pishmisheva M., Rangelova V., Stoyanova A., Yoncheva V. (2023). Age and Gender Trends in the Prevalence of Markers for Hepatitis E Virus Exposure in the Heterogeneous Bulgarian Population. Life.

[66] Jelicic P., Ferenc T., Mrzljak A., Jemersic L., Janev-Holcer N., Milosevic M., Bogdanic M., Barbic L., Kolaric B., Stevanovic V. (2022). Insights into Hepatitis E Virus Epidemiology in Croatia. World J. Gastroenterol..

[67] Miletić M., Vuk T., Hećimović A., Stojić Vidović M., Jemeršić L., Jukić I. (2019). Estimation of the Hepatitis E Assay-Dependent Seroprevalence among Croatian Blood Donors. Transfus. Clin. Biol..

[68] Vilibic-Cavlek T., Vilibic M., Kolaric B., Jemersic L., Kucinar J., Barbic L., Bagaric A., Stevanovic V., Tabain I., Sviben M. (2016). Seroepidemiology of Hepatitis E in Selected Population Groups in Croatia: A Prospective Pilot Study. Zoonoses Public. Health.

[69] Dalekos G.N., Zervou E., Elisaf M., Germanos N., Galanakis E., Bourantas K., Siamopoulos K.C., Tsianos E.V. (1998). Antibodies to Hepatitis E Virus among Several Populations in Greece: Increased Prevalence in an Hemodialysis Unit. Transfusion.

[70] Pittaras T., Valsami S., Mavrouli M., Kapsimali V., Tsakris A., Politou M. (2014). Seroprevalence of Hepatitis E Virus in Blood Donors in Greece. Vox Sang..

[71] Psichogiou M.A., Tassopoulos N.C., Papatheodoridis G.V., Tzala E., Klarmann R., Witteler H., Schlauder G.G., Troonen H., Hatzakis A. (1995). Hepatitis E Virus Infection in a Cohort of Patients with Acute Non-A, Non-B Hepatitis. J. Hepatol..

[72] Zervou E.K., Georgiadou S.P., Liapi G.K., Karabini F., Giogiakas V., Zisiadis K., Gatselis N.K., Goudevenos I., Dalekos G.N. (2005). Markers of Hepatitis Viruses and Human T-Lymphotropic Virus Types I/II in Patients Who Have Undergone Open-Heart Surgery: Evidence of Increased Risk for Exposure to HBV and HEV. Eur. J. Intern. Med..

[73] Aniţă A., Gorgan L., Aniţă D., Oşlobanu L., Pavio N., Savuţa G. (2014). Evidence of Hepatitis E Infection in Swine and Humans in the East Region of Romania. Int. J. Infect. Dis..

[74] Voiculescu M., Iliescu L., Ionescu C., Micu L., Ismail G., Zilisteanu D., Radasan A., Micu G., Pertache I. (2010). A Cross-Sectional Epidemiological Study of HBV, HCV, HDV and HEV Prevalence in the SubCarpathian and South-Eastern Regions of Romania. J. Gastrointestin Liver Dis..

[75] Delič D., Nešić Z., Žerjav S., Pešić I., Popović N., Simonović J. (2003). Hepatitis E Virus Infection in Serbia: Epidemiology and Clinical Features. Arch. Gastroenterohepatol..

[76] Petrović T., Lupulović D., Jiménez de Oya N., Vojvodić S., Blázquez A.-B., Escribano-Romero E., Martín-Acebes M.A., Potkonjak A., Milošević V., Lazić S. (2014). Prevalence of Hepatitis E Virus (HEV) Antibodies in Serbian Blood Donors. J. Infect. Dev. Ctries..

[77] Aksu K., Kabasakal Y., Sayiner A., Keser G., Oksel F., Bilgiç A., Gümüşdiş G., Doganavşargil E. (1999). Prevalences of Hepatitis A, B, C and E Viruses in Behçet’s Disease. Rheumatology.

[78] Akyüz F., Çavuş B., Pınarbaşı B., Bozacı M., Baran B., Akyuz U., Uyanıkoglu A., Demir K., Beşışık F., Özdil S. (2019). Cryptogenic Liver Cirrhosis and Hepatitis E Virus (HEV): Are They Related?. Ann. Hepatol..

[79] Aydin H., Uyanik M.H., Albayrak A., Özmen E., Aktaş O. (2013). Anti-HEV Seroprevalence in Blood Donors in Erzurum. Viral Hepatit Derg..

[80] Aydın N.N., Ergünay K., Karagül A., Pınar A., Us D. (2015). Investigation of the hepatitis E virus seroprevalence in cases admitted to Hacettepe University Medical Faculty Hospital. Mikrobiyol. Bul..

[81] Çakmak-Topfedaisi Ö., Şener A. (2020). Seroprevalence of Hepatitis E in Hospital Employees and Investigation of Risk Factors. Klimik Derg..

[82] Cengiz K., Ozyilkan E., Coşar A.M., Günaydin M. (1996). Seroprevalence of Hepatitis E in Hemodialysis Patients in Turkey. Nephron.

[83] Cevrioglu A.S., Altindis M., Tanir H.M., Aksoy F. (2004). Investigation of the Incidence of Hepatitis E Virus among Pregnant Women in Turkey. J. Obstet. Gynaecol. Res..

[84] Ceylan A., Ertem M., Ilcin E., Ozekinci T. (2003). A Special Risk Group for Hepatitis E Infection: Turkish Agricultural Workers Who Use Untreated Waste Water for Irrigation. Epidemiol. Infect..

[85] Eker A., Tansel O., Kunduracilar H., Tokuç B., Yuluğkural Z., Yüksel P. (2009). Hepatitis E virus epidemiology in adult population in Edirne province, Turkey. Mikrobiyol. Bul..

[86] Köksal I., Aydin K., Kardes B., Turgut H., Murt F. (1994). The Role of Hepatitis E Virus in Acute Sporadic Non-A, Non-B Hepatitis. Infection.

[87] Olcay D., Eyigün C.P., Özgüven Ş.V., Avci I.Y., Beşirbellioǧlu A.B., Tosun S.Y., Pahsa A. (2003). Anti-HEV Antibody Prevalence in Three Distinct Regions of Turkey and Its Relationship with Age, Gender, Education and Abortions. Turk. J. Med. Sci..

[88] Sezgin O., Yaraş S., Tezcan Ülger S., Aslan G., Tiftik E.N. (2021). The Prevalence of Hepatitis E Virus Infection in the Adult Turkish Population: A Systematic Review of the Literature and Prevalence Study in Blood Donors in Mersin Province. Turk. J. Gastroenterol..

[89] Thomas D.L., Mahley R.W., Badur S., Palaoglu K.E., Quinn T.C. (1993). Epidemiology of Hepatitis E Virus Infection in Turkey. Lancet.

[90] Yaşar O., Karatayli E., Cengiz G., Kızılpınar M., Yurdcu E., Albayrak R., Güven A., Arslan Ö., Karahan C., Otlu B. (2019). HEV Seroprevalence in Blood Donors in Turkey by Two Commercial Total Anti-HEV Ab ELISA Kits. J. Med. Virol..

[91] Teoharov P., Tiholova M., Draganov P., Lilyanova V., Ivanova R., Varleva T. (1995). First Cases of Hepatitis E Virus Infection in Bulgaria. Infectology.

[92] Corneillie L., Mézière L., Montpellier C., Drouet B., Aliouat-Denis C.-M., Cocquerel L. (2026). Update on the Molecular and Cellular Biology of Hepatitis E Virus and Therapeutic Opportunities. Antivir. Res..

[93] Vesković Moračanin S.M., Kureljušić B.I., Maletić J., Kureljušić J.M., Jezdimirović N.V., Vasić A.M., Milovanović B.Z., Savić B.M. (2026). Hepatitis E in Wildlife: Emerging Threats to Human Health. Vet. Sci..

[94] Sadik S., van Rijckevorsel G.G.C., van Rooijen M.S., Sonder G.J.B., Bruisten S.M. (2016). Seroprevalence of Hepatitis E Virus Differs in Dutch and First Generation Migrant Populations in Amsterdam, the Netherlands: A Cross-Sectional Study. BMC Infect. Dis..

[95] Tene S.D., Diouara A.A.M., Sané S., Coundoul S. (2025). Hepatitis E Virus (HEV) Infection in the Context of the One Health Approach: A Systematic Review. Pathogens.

[96] Tsachev I., Gospodinova K., Pepovich R., Takova K., Kundurzhiev T., Zahmanova G., Kaneva K., Baymakova M. (2023). First Insight into the Seroepidemiology of Hepatitis E Virus (HEV) in Dogs, Cats, Horses, Cattle, Sheep, and Goats from Bulgaria. Viruses.

[97] Demirci M., Yiğin A., Ünlü Ö., Kılıç Altun S. (2019). Detection of HEV RNA amounts and genotypes in raw milks obtained from different animals. Mikrobiyol. Bul..

[98] Dziedzinska R., Krzyzankova M., Bena M., Vasickova P. (2020). Evidence of Hepatitis E Virus in Goat and Sheep Milk. Viruses.

[99] Kokkinos P., Kozyra I., Lazic S., Bouwknegt M., Rutjes S., Willems K., Moloney R., de Roda Husman A.M., Kaupke A., Legaki E. (2012). Harmonised Investigation of the Occurrence of Human Enteric Viruses in the Leafy Green Vegetable Supply Chain in Three European Countries. Food Environ. Virol..

[100] Mrzljak A., Dinjar-Kujundzic P., Jemersic L., Vilibic-Cavlek T. (2021). The Burden of Hepatitis E Infection in Chronic Liver Diseases in Croatia. Vector Borne Zoonotic Dis..

[101] Rivadulla E., Varela M.F., Mesquita J.R., Nascimento M.S.J., Romalde J.L. (2019). Detection of Hepatitis E Virus in Shellfish Harvesting Areas from Galicia (Northwestern Spain). Viruses.

[102] Okamoto H. (2026). Shellfish as a Potential Source of Hepatitis E Virus: Epidemiological Evidence, Biological Plausibility, and Research Gaps. Viruses.

[103] Kanai Y., Miyasaka S., Uyama S., Kawami S., Kato-Mori Y., Tsujikawa M., Yunoki M., Nishiyama S., Ikuta K., Hagiwara K. (2012). Hepatitis E Virus in Norway Rats (Rattus Norvegicus) Captured around a Pig Farm. BMC Res. Notes.

[104] Xu W.-T., Ding Y.-S., Chen Y.-G., Feng Y.-H., Li J., Jin J.-G., Wei X.-N., Wu F., Wang X.-Y., Dang X.-T. (2026). Elevated Risk of Hepatitis E Virus Infection among Sheep Smallholders in Xinjiang, China. One Health.

[105] Diehl T.M., Adams D.J., Nylund C.M. (2018). Ingesting Self-Grown Produce and Seropositivity for Hepatitis E in the United States. Gastroenterol. Res. Pract..

[106] Houcine N., Jacques R., Salma F., Anne-Gaëlle D., Amin S., Mohsen H., Hamadi B., Christophe R., Patrice A., Mahjoub A. (2012). Seroprevalence of Hepatitis E Virus Infection in Rural and Urban Populations, Tunisia. Clin. Microbiol. Infect..

[107] Aydin H., Uyanik M.H., Karamese M., Timurkan M.O. (2016). Seroprevalence of Hepatitis E Virus in Animal Workers in Nonporcine Consumption Region of Turkey. Future Virol..

[108] Bayram A., Eksi F., Mehli M., Sözen E. (2007). Prevalence of Hepatitis E Virus Antibodies in Patients with Chronic Hepatitis B and Chronic Hepatitis C. Intervirology.

[109] Cesur S., Akin K., Doğaroğlu I., Birengel S., Balik I. (2002). Hepatitis A and hepatitis E seroprevalence in adults in the Ankara area. Mikrobiyol. Bul..

[110] Gorski I., Babić I., Bingulac-Popović J., Topić-Šestan P., Jagnjić S., Jemeršić L., Prpić J., Jukić I. (2023). Prevalence of HEV RNA in Croatian Blood Donors. Transfus. Clin. Biol..

[111] Mladenova-Dimitrova Z., Gotseva A., Velcheva D. (2020). Prevalence of Hepatitis E in General Population in Bulgaria, 2017-2019. Gen. Med..

[112] Psichogiou M., Tzala E., Boletis J., Zakopoulou N., Loutradi A., Maliori M., Kourea-Kremastinou J., Stratigos J., Hatzakis A. (1996). Hepatitis E Virus Infection in Individuals at High Risk of Transmission of Non-A, Non-B Hepatitis and Sexually Transmitted Diseases. Scand. J. Infect. Dis..

[113] Psichogiou M., Vaindirli E., Tzala E., Voudiclari S., Boletis J., Vosnidis G., Moutafis S., Skoutelis G., Hadjiconstantinou V., Troonen H. (1996). Hepatitis E Virus (HEV) Infection in Haemodialysis Patients. The Multicentre Haemodialysis Cohort Study on Viral Hepatitis. Nephrol. Dial. Transplant..

[114] Teoharov P., Kevorkyan A., Raycheva R., Golkocheva-Markova E., Trandeva-Bankova D., Andonov A. (2014). Data on the Prevalence of Hepatitis E Virus in Bulgaria. Comptes Rendus L’Academie Bulg. Sci..

[115] Thomas D.L., Mahley R.W., Badur S., Palaoglu E., Quinn T.C. (1994). The Epidemiology of Hepatitis C in Turkey. Infection.

[116] Yaraş S., Özdoğan O., Tezcan Ülger S., Aslan G., Tiftik E.N., Sezgin O. (2025). Hepatitis E Virus (HEV) Seroprevalence in Cryptogenic Cirrhosis: From Evidence of High Frequency to the Impact on Disease Progression. Medicina.

